# Single-asperity contact mechanics with positive and negative work of adhesion: Influence of finite-range interactions and a continuum description for the squeeze-out of wetting fluids

**DOI:** 10.3762/bjnano.5.50

**Published:** 2014-04-08

**Authors:** Martin H Müser

**Affiliations:** 1 Jülich Supercomputing Centre, FZ Jülich, 52428 Jülich, Germany

**Keywords:** cohesive zone model, contact mechanics, environmental, fluid squeeze-out, nanomechanics, single-asperity contacts

## Abstract

In this work, single-asperity contact mechanics is investigated for positive and negative work of adhesion Δγ. In the latter case, finite-range repulsion acts in addition to hard-wall constraints. This constitutes a continuum model for a contact immersed in a strongly wetting fluid, which can only be squeezed out in the center of the contact through a sufficiently large normal load *F*_N_. As for positive work of adhesion, two stable solutions can coexist in a finite range of normal loads. The competing solutions can be readily interpreted as contacts with either a load-bearing or a squeezed-out fluid. The possibility for coexistence and the subsequent discontinuous wetting and squeeze-out instabilities depend not only on the Tabor coefficient μ_T_ but also on the functional form of the finite-range repulsion. For example, coexistence and discontinuous wetting or squeeze-out do not occur when the repulsion decreases exponentially with distance. For positive work of adhesion, the normal displacement mainly depends on *F*_N_, Δγ, and μ_T_ but – unlike the contact area – barely on the functional form of the finite-range attraction. The results can benefit the interpretation of atomic force microscopy in liquid environments and the modeling of multi-asperity contacts.

## Introduction

The continuum description of single-asperity contact mechanics has received much attention in the last few decades. This is in large parts because it describes force-displacement curves rather accurately down to nanometer scales relevant to atomic force microscopy (AFM) [1–3]. The contributions to the linear elasticity of (frictionless) single-asperity contacts most central to this work are the following: Hertz [4] solved the contact mechanics of a parabolic tip pressed against a substrate for hard-wall repulsion. He found that the contact area *A*_c_ and the separation between the two solids *d* both disappear continuously with 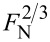 as the normal load squeezing the two solids together, *F*_N_, approaches zero. Derjaguin, Muller, and Toporov (DMT) [5] included adhesion as a long-range interaction, in which case adhesion effectively acts as a normal load that is independent of the contact-radius *a*_c_. Johnson, Kendall, and Roberts (JKR) [6] solved the problem for short-range adhesion, for which the attractive surface forces act directly at the contact line. Unlike Hertz and DMT, JKR found finite values for *A*_c_ and *d* at pull off. Tabor [7] introduced a dimensionless parameter, μ_T_, now known as Tabor coefficient, allowing one to estimate if a contact is closer to DMT or to JKR theory. He actually recognized that DMT and JKR describe the opposite limits of long- and short-range forces, respectively. This had not been known before but was soon confirmed in numerical simulations by Muller, Yushenko, and Derjaguin [8]. Lastly, Maugis [9] used a cohesive-zone model introduced by Dugdale (MD) and found analytical solutions for intermediate-range adhesion at arbitrary values of μ_T_.

Although single-asperity, linearly-elastic, adhesive contacts mechanics is a rather mature field [10], two key issues remain worth addressing: First, only few studies have considered the case of negative work of adhesion [11–12], Δγ < 0, specifically finite-range repulsion between two surfaces acting in addition to hard-wall repulsion. In particular, the concept of the Tabor coefficient has not yet been applied to negative Δγ. Therefore, I investigate if there are different regimes for Δγ < 0 as is the case for contacts with Δγ > 0, which are classified as JKR for large μ_T_ and as DMT for small μ_T_. This includes a study of which parameters determine the behavior near squeeze-out as well as a comparison to the behavior near pull-off for Δγ > 0. For the latter, it is straightforward to deduce from established results how the *a*_c_(*F*_N_) relation depends on the Tabor coefficient in the DMT and the JKR limit. Specifically, *a*_c_ − *a*_p_ 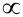 (*F*_N_ + *F*_p_)^κ^ for *F*_N_ ≥ −*F*_p_, where *F*_p_ and *a*_p_ are pull-off force and pull-off radius, respectively. They both depend on μ_T_ just like the exponent κ, e.g., *a*_p_ > 0 and κ = 1/2 in the JKR limit, while *a*_p_ = 0 and κ = 1/3 for DMT. In the present comparison of squeeze-out (finite-range repulsion) versus pull-off (finite-range attraction), I also study whether the exponent κ changes continuously between JKR and DMT or discontinuously – as assumed implicitly in the Carpick–Ogletree–Salmeron (COS) model [1].

The second motivation for this paper is that it has not yet been investigated sufficiently how the (precise) functional form for adhesive interactions affects contact mechanics – assuming that all continuum parameters, from normal load to Tabor coefficient, are identical. It is only established that there is little sensitivity in the limits of large and zero Tabor coefficients. Yet, when studying contact-mechanics between macroscopic, adhesive, rough surfaces in the context of continuum-mechanics, one would want to know how to best reach the JKR limit, which appears to be the relevant limit for that application. For example, it is used implicitly in Persson theory for nominally flat, adhesive surfaces [13]. In fact, the current work was initiated by the desire to add adhesion into a Green’s function molecular dynamics (GFMD) code used to model the contact between rough surfaces. To model adhesion, one needs to identify a functional form for the finite-range surface forces allowing one to reach the JKR limit in an efficient and well-controlled fashion. It quickly became clear that doing a clean job is not entirely trivial and that modeling single-asperity contacts ought to be better understood first and moreover is interesting in its own right.

The remainder of this paper is organized as follows: I first introduce the model, sketch the numerical methods and discuss difficulties arising in simulations in the limit of large and small Tabor coefficients. Next, I present a brief dimensional analysis motivating the commonly used unit system and the Tabor coefficient. The result section opens with adhesive contacts. There, I reproduce some established results and investigate how sensitive results are on the details of the interaction model. That section also contains a comparison to and an asymptotic analysis of the MD model motivating some minor modifications of the empirical COS equations [1]. Next, results on positive adhesion are presented before the major results are summarized and conclusions are drawn.

## Results and Discussion

### Definition of the model

**Figure 1:**
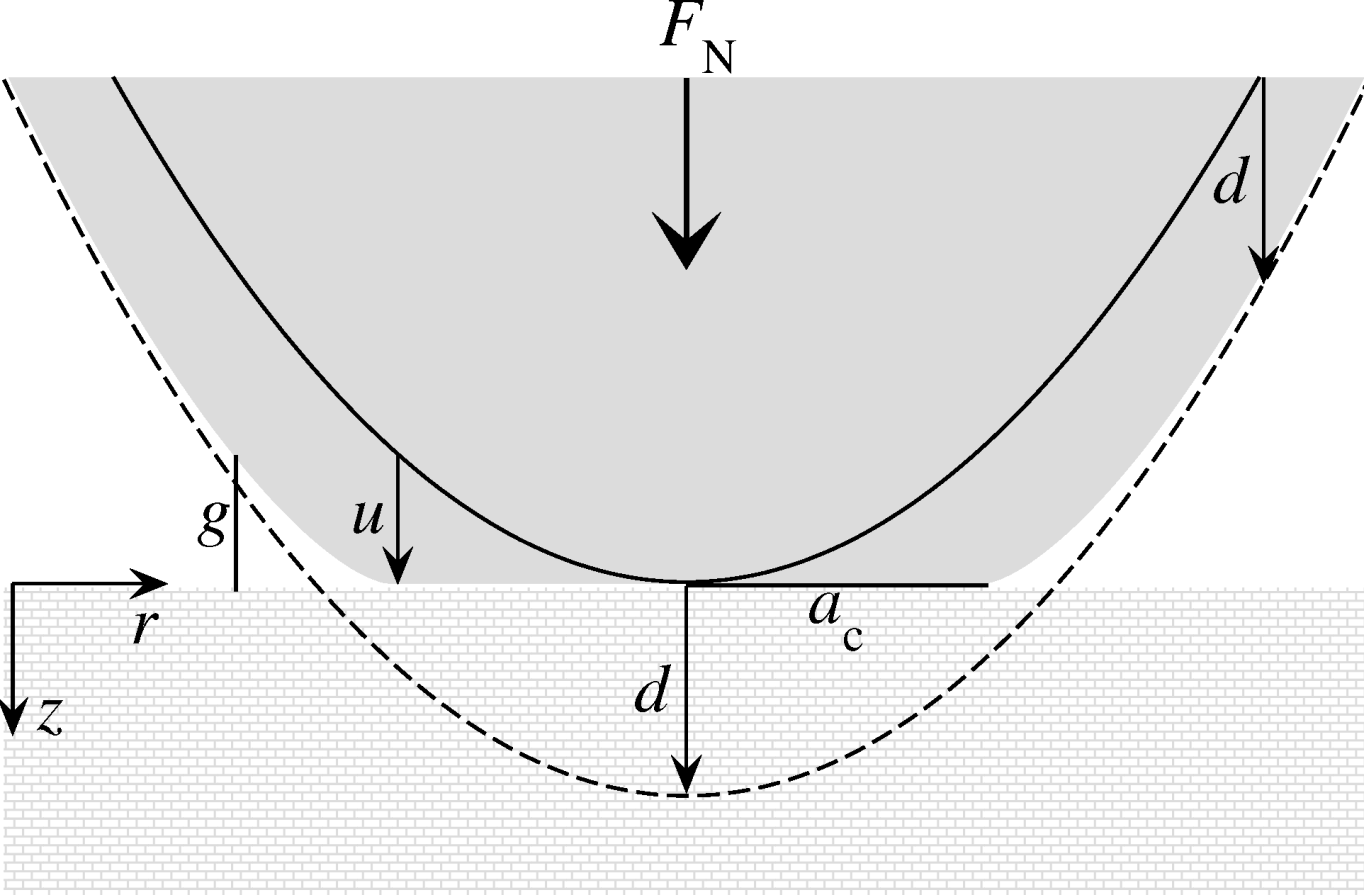
Geometry of the deformed tip (upper grey solid), the substrate (lower solid), and the reference tip (solid line). The dotted line represents a hypothetical tip that is allowed to penetrate the substrate the distance *d* into the substrate without deforming. The following vectors are introduced in the sketch: Normal load *F*_N_ acting on the center of mass of the tip, the elastic displacement field *u*, and the displacement *d* of the tip’s center of mass. In addition, two scalar quantities, namely the contact radius *a*_c_ and the gap (field) *g* are shown.

In this section, the single-asperity contact problem is introduced. As shown in Figure 1, we consider a stiff, ideally-flat wall positioned in the *xy* plane at *z* = 0 and a linearly-elastic tip of parabolic shape. Its undeformed surface is given by

[1]
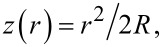


where *R* is the radius of curvature and 
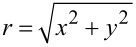
 the in-plane distance of the center of the tip from the origin of the coordinate system. The elastic displacement of the tip, *u*(*x,y*) is formally a function of both in-plane coordinates, although the equilibrium solutions only depend on *r*. The gap *g*(*x,y*) indicates the distance between the deformed tip and the substrate, i.e.,

[2]
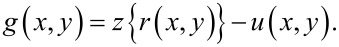


It is furthermore assumed that the tip cannot penetrate the substrate. This can be stated as a non-holonomic boundary condition

[3]
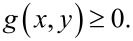


Alternatively, one can formally introduce a short-range, hard-wall repulsion [14]

[4]
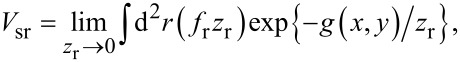


where *f*_r_ is an arbitrary positive constant of unit force per area. Note that the integrand on the r.h.s. of Equation 4 is zero for finite gaps while it diverges for negative gaps. Depending on the problem, it can be more convenient to use either the non-holonomic boundary condition or the energy-based description of the short-range repulsion.

This work also considers finite-range adhesive or finite-range repulsive interactions *V*_fr_, which only depend on the local gap. The default expression for it is:

[5]
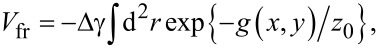


where Δγ is the work of adhesion per surface area that is obtained when a flat tip touches the substrate in a continuum description. The choice of the functional dependence of *V*_fr_ is not motivated by the true functional form for the interactions between any two real solids, but for the moment being, it is a matter of convenience. Alternative interaction models for the integrand are introduced in a seperate section.

An important property of all models for *V*_fr_ is that the interaction is characterized by a prefactor representing the work of adhesion and a single length scale *z*_0_. The choice of the latter allows one to localize adhesive stress near the contact line through *z*_0_ → 0 or to extend the adhesive interactions to radii much exceeding the contact radius *a*_c_ for *z*_0_ → ∞. Of course, *z*_0_ can take any value between zero and infinity so that intermediate-range interactions can be modeled as well. Note that *V*_fr_ and *V*_sr_ are qualitatively different: The prefactor of the short-range potential is formally zero, because *f*_r_ is finite and *z*_r_ very small. In other words, *z*_r_ represents the size of an “infinitesimally-small” atom whose size is irrelevant on a continuum scale. In contrast, the prefactor of the finite-range potential is considered finite as well as the range of interaction *z*_0_. It represents a “collective” length scale, such as the decay length of density oscillations in the fluid [15] or the radius of gyration of a polymer.

The displacement *u*(*x,y*) and other fields (gap and stress) will be expressed not only in real space, but also in Fourier space. This is done most conveniently by using in-plane periodic boundary conditions. The respective boundaries lie at *x* or *y* = ±*L*/2, which should be chosen such that *L* (the linear dimension of the simulation cell) is much greater than the linear dimension of the contact zone. The latter includes the contact and the area of non-negligible adhesive (or finite-range repulsive forces) stresses. The following convention for the Fourier transform shall be employed

[6]
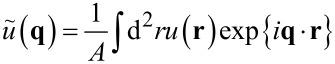


[7]
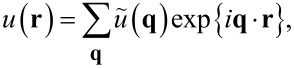


where the wave vector components satisfy *q*_α_ = 2π*n*/*L*, *A* = *L*^2^ is the integration domain, and *n* an integer number. With these definitions, one can express the elastic energy of the deformed tip (in the small-slope approximation) as

[8]
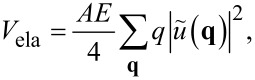


where *E* is the effective modulus, *E* = *E*_Y_/(1 − ν^2^), *E*_Y_ being the Young’s modulus and ν the Poisson ratio. The convention of using the symbol E^*^ for the effective modulus is abandoned for clarity, because primes will be used later to indicate scaled coordinates and scaled parameters.

Since 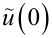 can be interpreted as the center of mass mode, the effect of a load (or normal force) exerted on the tip leads to an energy

[9]
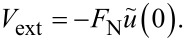


When solving the contact problem, one seeks to minimize the total energy

[10]
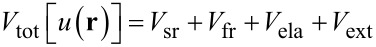


with respect to *u*(*x,y*), i.e., the solution *u*_0_(**r**) must satisfy

[11]
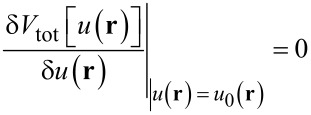


for each **r**. Here, δ indicates a functional derivative. In a discrete representation of the problem, **r** is an index so that the functional derivative in Equation 11 has to be replaced by a partial derivative.

#### Alternative interaction models

Throughout this paper, different functional forms for the finite-range interactions between surfaces are considered in addition to the “default” or “exponential” model introduced in Equation 5. Functions similar to the ones used in this work have already been employed for the simulation of mode I fracture or delamination. Depending on the authors, the functions are called the cohesive zone model [16], the crack evolution function [17], or the traction-separation relation [18]. However, it is not clear how the results obtained for mode I fracture geometries relate to Hertzian contacts. This is the main reason why the results obtained within the cohesive zone model cannot be compared in a straightforward fashion to those of the current study.

**Figure 2:**
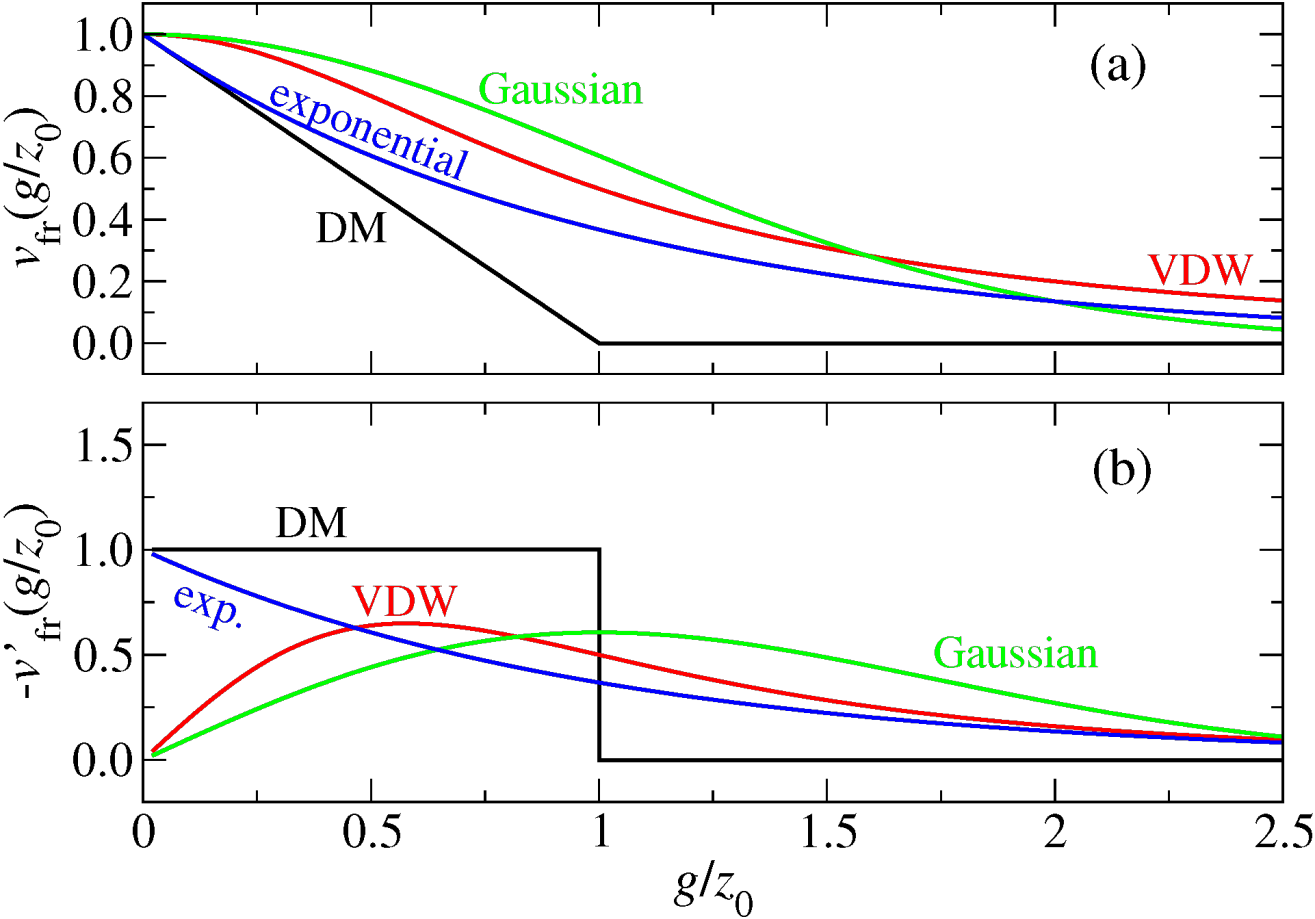
(a) Finite-range surface energies and (b) forces per unit area for the models investigated in this study.

The additional models in the current work replace the integrand on the r.h.s. of Equation 5 with the following expressions:

[12]
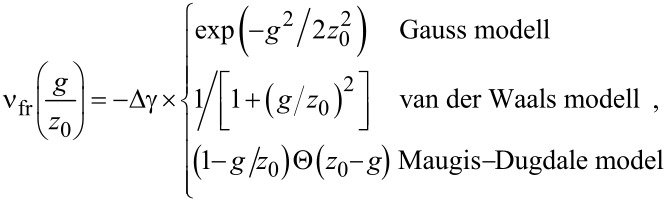


where Θ(···) denotes the Heavyside step functions, which is one for positive arguments and zero otherwise. The interaction potentials and their first derivatives are shown in Figure 2.

All expressions take the same value, −Δγ, for the adhesive energy when the two surfaces touch, i.e., in each case the work of adhesion is Δγ. In this sense, all four models produce the same continuum limit. However, in two models, namely the Gauss and the van der Waals (VDW) integrands, the derivative 
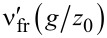
 goes to zero when the two surfaces touch, while it remains finite in the exponential model and the MD model.

As stated before, none of the models are supposed to be highly realistic representations of surface forces, although each model may have its justification. In particular the exponential model follows from the argument that long-range density correlations in fluids are governed by a single length [15]. In a high-density fluid, the correlation length becomes complex [15], which then leads to layering transitions as discussed recently [19]. The VDW model might approximate the long-range van der Waals interactions in a way that Δγ reflects the Hamaker constant. Depending on the confined system in question, other effective interactions might be possible. However, all models represent the feature that surfaces repel upon close approach (i.e., when atoms from opposing surfaces bump into each other, which is implemented through the hard-wall repulsion) and that attraction – or additional repulsion – may occur at finite distance. Continuous short-range repulsive forces are not used here. Doing so would complicate the definition of contact and thus contact radius, which has remained controversial for systems without hard-wall or hard-disk interactions [20–23]. Lastly, the equations to be solved would become very stiff and thus the simulation inefficient if the hard-wall constraint was replaced by potentials with large curvature.

In the context of the squeeze-out of fluids, the MD and the exponential model might not be physically meaningful for small ratios of *g*/*z*_0_: when one flat solid is placed on top of another flat solid with an infinitesimally small external load (in the absence of a fluid), the two solids would repel, although they “cannot know”, away from a contact line, that a fluid wants to penetrate. Nonetheless, the exponential model has been used in early study of squeeze-out of fluids [12]. Forces between two (flat) surfaces in the Gauss and the VDW model are zero either for intimate mecanical contact or at infinite separation.

#### Dimensional analysis

In this section, I present a simple dimensional analysis of the contact problem. The result of the analysis is a meaningful set of units, which, in similar form, has already been established by Maugis [9]. However, in the present analysis, units are not motivated from the solutions but rather straight from the beginning, i.e., by the expressions defining the model. This is why Maugis’ and the present units differ by dimensionless prefactors, which, however, always turn out close to unity. In the subsequent derivation, it is not necessary to know the precise functional dependence of the finite-range forces.

Assume we know the solution *u*_0_(**r**) minimizing *V*_tot_ for a given set of parameters defining our model, i.e., *u*(**r**) solves the contact problem for a specific set of values for *E, R*, *F*_N_, Δγ, and *z*_0_. It is then possible to “recycle” the shape of the function *u*_0_(**r**) to solve a different problem defined by different parameters *E*′, *R*′, 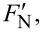 Δγ′, and 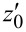. Specifically, if each individual summand of 
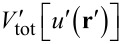
 is identical to the equivalent term in *V*_tot_[*u*(**r**)] (up to a multiplicative constant, which can be set to one), then 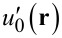 minimizes 
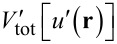
 given that *u*_0_(**r**) minimizes *V*_tot_[*u*(**r**)].

The transformation, 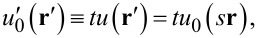 in which in-plane coordinates are scaled as **r**′ ≡ *s***r** and normal coordinates are scaled as *z*′ = *tz* leaves the shape of the solution unchanged. Of course, *z*(**r**) and thus *g*(**r**) must be transformed the same way as *u*(**r**). Therefore, the radius of curvature of the “new” tip is *R*′ = *s*^2^*R*/*t*.

Let us investigate how one has to alter each individual term contributing to 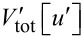 so that it matches its equivalent in *V*_tot_[*u*]. (i) The hard-wall repulsive energy *V*_fr_ is unproblematic. It disappears for the old and the new solution, because of the limit *z*_r_ → 0, i.e., 
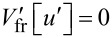
. (ii) To recycle the *V*_fr_ calculation, we need to set 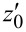 = *tz*_0_. Keeping in mind that *A*′ = *s*^2^*A*, where *A* = *L*^2^ is the original integration domain, it follows that 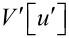 = *s*^2^(Δγ′/Δγ)*V*[*u*]. (iii) For the calculation of the elastic energy, it is useful to keep in mind that *q*′ = *q*/*s* and that *A*′ = *s*^2^*A*. This means that 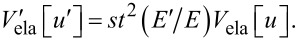 (The integer indices enumerating the wave vectors are identical for the original and the new domain.) (iv) Lastly, the load-related energy becomes 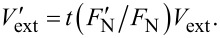 In summary, we can recycle our solution with the following substitutions

[13]
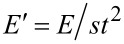


[14]
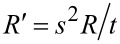


[15]
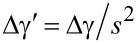


[16]
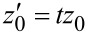


[17]
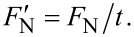


Let us first consider the case of no external force, *F*_N_ = 0, so that we investigate the “intrinsic” system properties. If we use *E* as the unit of pressure, which is done until further notice, all three remaining parameters defining the system can be expressed to be of unit length, i.e, *R, z*_0_, and Δγ/*E*. Wether a potential should be classified as short- or long-ranged can only depend on a non-dimensionalized interaction length. This means that *z*_0_ has to be expressed in the two remaining units of length (*R* and Δγ/*E*) such that the dimensionless ratio

[18]
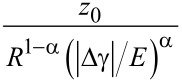


remains unchanged under a scaling transformation.

Let us now chose the radius of curvature as the unit of length, or, alternatively, consider only those scaling transformations that leave *R* constant. This can be achieved by setting *t* = *s*^2^, which maps an infinite parabola (*x* → *z* = *x*^2^) onto itself (*sx* → *s*^2^*z*). I note in passing that such a transformation might not be meaningful for a scaling analysis of the contact mechanics of randomly rough surfaces, which will be presented elsewhere.

Inserting the relevant equalities from Equation 13–Equation 17 reveals that choosing α = 2/3 leaves the expression in Equation 18 constant. As a consequence, the range of influence of the adhesive term is best quantified by the term

[19]
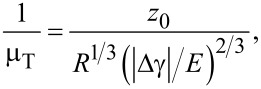


where μ_T_ is known as Tabor coefficient – up to a dimensionless, multiplicative constant. It remains invariant under all scaling transformation in Equation 13–Equation 17 leaving the radius of curvature unchanged.

From Equation 19 one can see that we need to send *z*_0_ → *w*^2/3^*z*_0_ in order to keep the Tabor coefficient constant when changing Δγ/*E* at constant *R* to *w*Δγ/*E*. This in turn implies a transformation of *x* → *w*^1/3^*x* for the in-plane coordinates, because *R* is supposed to remain unchanged. It follows that *a*_c_(*F*_N_ = 0) → *w*^1/3^*a*_c_(*F*_N_ = 0) and thus *a*_c_ 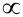 (|Δγ|/*E*)^1/3^. The unit of *a*_c_ can be fixed by multiplying the r.h.s. of this proportionality with *R*^2/3^. Otherwise the proportionality coefficient can only depend on μ_T_, and of course, on the sign of Δγ. Therefore, we can write

[20]
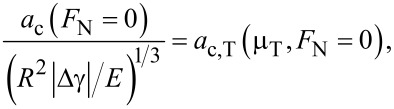


such that the r.h.s. of the equation only depends on the Tabor coefficient and the functional form of the surface interaction. Since we have not used the explicit functional form of our default surface interaction (other than that it depends only on a single length scale), the conclusions drawn in this section extends to any choice for *V*_fr_ considered in this work.

To include finite loads into the analysis, note that the ratio *F*_N_/|Δγ|*R* does not change under the transformation Equation 13–Equation 17. This allows us to express a properly undimensionalized contact radius as a dimensionless function of a properly dimensionless load

[21]
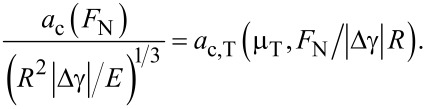


From this last equation, it also becomes clear that the pull-off (or the squeeze-out) force is proportional to |Δγ|*R*, i.e., by identifying the value of *F*_N_/|Δγ|*R* at which the function *a*_c,T_ takes its minimum value. Therefore, it is most meaningful to normalize the force with Δγ*R*, unless, of course, Δγ = 0.

**Figure 3:**
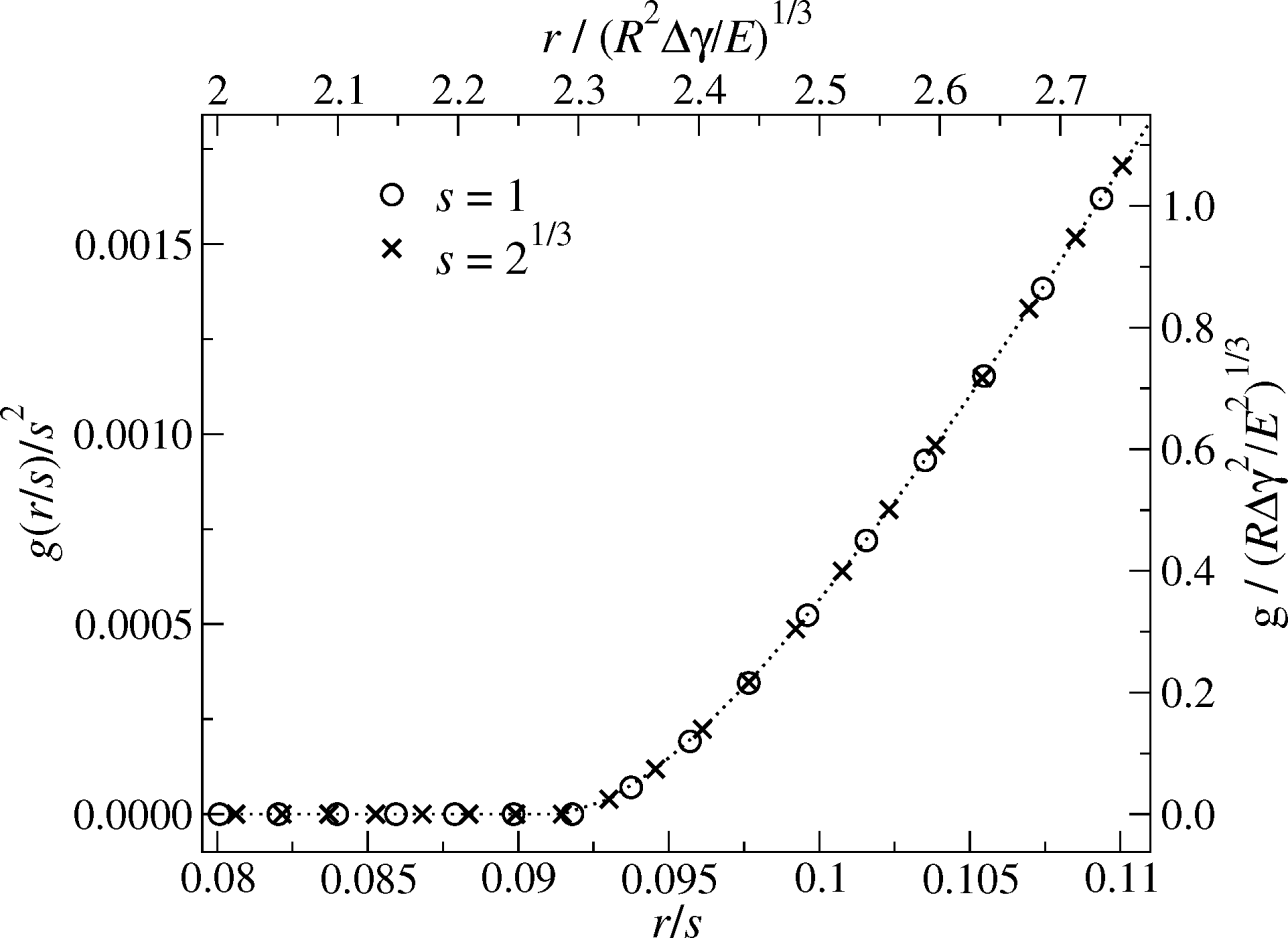
Scaled gap *g*(*r*) as a function of scaled distance from origin *r* for two different parameter realizations related through the scaling transformation Equation 13–Equation 17. Parameters used are *E* = *L* = μ_T_ = 1, *F*_N_ = 1·10^−4^/*w* and Δγ = 0.64·10^−4^/*w*. The surface is discretized into 512 × 512 elements. Circles *s* = 1 with *w* = 1 → *z*_0_ = 0.0016, crosses *w* = 1/2 → *z*_0_ = 2^2/3^ · 0.0016. For the second data set, this implies a scaling factor of *t* = 2^2/3^ for variables linear in normal coordinates and thus *s* = 2^1/3^ for variables proportional to in-plane coordinates. The units on the normal side of the axes are in “absolute” units, i.e., *L* = 1. The units on the opposite axes correspond to those that remain invariant under a scaling transformation. The dotted line is drawn to guide the eye.

The approach is validated in Figure 3. It shows the spatial dependence of the gap for two different parameterizations. Small deviations, which are not visible to the naked eye, occur. They are due to finite-size and discretization effects. For example, the ratio *a*_c_/*L* is not exactly zero and takes different values for different values of *s* for a fixed number of points used to represent the elastic surface.

The normal displacement can be nondimensionalized in a similar fashion as the contact radius, except that it needs to be rescaled with the factor *w*^2/3^ rather than with *w*^1/3^. This is why it must obey

[22]
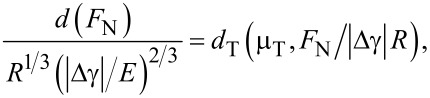


where all terms on the r.h.s. of the equation are again dimensionless, while those on the l.h.s. are allowed to have units. Thus, displacements and gaps are best represented in units of *R*^1/3^(|Δγ|/*E*)^2/3^, while contact radii are more meaningfully expressed as multiples of *R*^2/3^(|Δγ|/*E*)^1/3^. As a result, numbers turn out of order unity when data is represented in these units, unless *F*_N_ approaches the pull-off threshold or distinctly exceeds Δγ*R*.

I conclude this section by summarizing the units used in this work and discuss some of the consequences arising from it: Specifically, the following units are used for:

[23]
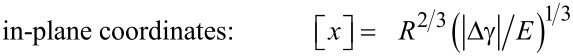


[24]
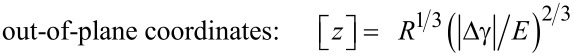


[25]
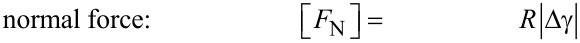


[26]
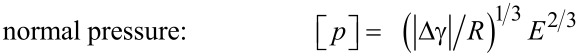


This list includes a “new” unit of normal stress or pressure, [*p*], which must be chosen proportional to [*F*_N_]/[*x*]^2^ rather than to *E* so that the regular definition of pressure applies. As noted above, *E* drops out of the definition of the unit for the normal force, implying that pull-off or squeeze-out forces cannot be functions of *E*. Instead they must equal *R*|Δγ| times dimensionless expressions that can only depend on μ_T_, and, of course, on the functional form of the interaction potential. In our units, the well-known *a*_c_(*F*_N_,E^*^,*R*,Δγ) relations can be simplified to

[27]
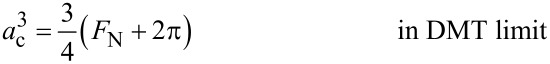


[28]
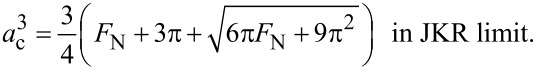


Hertzian contact mechanics is obtained in either limit for *F*_N_ >> 1. Finally, note that Maugis’ choice for units slightly differs from ours in that he used πΔγ rather than Δγ in Equation 23–Equation 26 and 3*E*/4 instead of *E*. The conversion between Maugis’ and our system is summarized in Equation 29–Equation 30.

#### Numerical analysis

Different methods can be used in the numerical solution of Equation 11. For the present study, Green’s function molecular dynamics (GFMD) [24] as described in [25] was employed. The only modification is the implementation of conservative surface forces acting in addition to the boundary condition *g*(**r**) ≥ 0. Moreover, the results in this work were produced with a serial code with typical run times of a few minutes. I refer to the literature for more details on GFMD [24–25]. Irrespective of the employed code or method, particular precautions, which are worth discussing, need to be taken into account when including adhesion or finite-range repulsion.

When simulating Hertzian contact mechanics, one needs to ensure that the discretization of the lattice Δ*a* satisfies Δ*a* << *a*_c_. Of course, methods based on spatially varying grids only need to obey that relation near the contact line. In addition, one wants *a*_c_ to be much less than the size of the simulation cell, at least in Fourier-based methods, such as GFMD. One then has the sequence of inequalities Δ*a* << *a*_c_ << *L*. In Hertzian contact mechanics, this is easy to achieve: choosing the discretization such that Δ*a*/*a*_c_ = 1/32 and *a*_c_/*L* = 1/8 already gives accurate results for the contact area, that is, to within less than 0.1% error if the contact area is determined through a fit of the radial pressure profile.

When including adhesion, an additional length enters, namely that associated with the adhesive zone. The additional adhesive radius or skin *a*_a_ then needs to be taken into consideration. When the Tabor coefficient is very small, *a*_a_ becomes large, and one needs *a*_a_ to lie within the simulation cell. A new series of inequalities is obtained: Δ*a* << *a*_c_ << *a*_a_ << *L*. If the normal stress changes smoothly with the gap, i.e., for long-range adhesion, the forces couple predominantly to large wavelength modes. This then results in a simple offset force, as is well known from DMT theory. However, numerical demands can become significant when *a*_c_ disappears continuously with decreasing load as in the DMT scenario. The condition *a*_c_ << *a*_a_ then becomes difficult to satisfy.

In the opposite case of a large Tabor coefficient, *a*_a_ is very small, potentially much smaller than *a*_c_. We still need to resolve the adhesive zone sufficiently well, because the stress has to be smooth on the given discretization. One thus obtains the series of inequalities *a* << *a*_a_ << *a*_c_ << *L*. In either limit of large or small Tabor coefficient, another inequality needs to be satisfied in addition to those for Hertzian contact mechanics.

While the contact area converges reasonably quickly as the respective inequalities are obeyed, the center-of-mass displacement *d*, which corresponds to 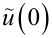 or *u*(*r* → ∞) only converges slowly. The reason is that the displacement field induced at a given point due to an external force only decays with the inverse distance from that point, i.e.,

[31]
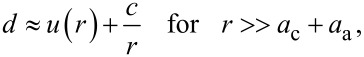


where *c* is a load- and system-dependent constant. Outside of the adhesive zone, this relation can be used, in principle, to extrapolate from finite *r* to infinite *r*, i.e., by determining *c* and *d* from two measurements taken sufficiently far outside the adhesive zone. In practice, this turns out problematic, because the periodic boundary conditions suppress the 1/*r* corrections near the boundaries.

For Hertzian contacts, it is still possible to use a slightly modified (empirical) correction

**Figure 4:**
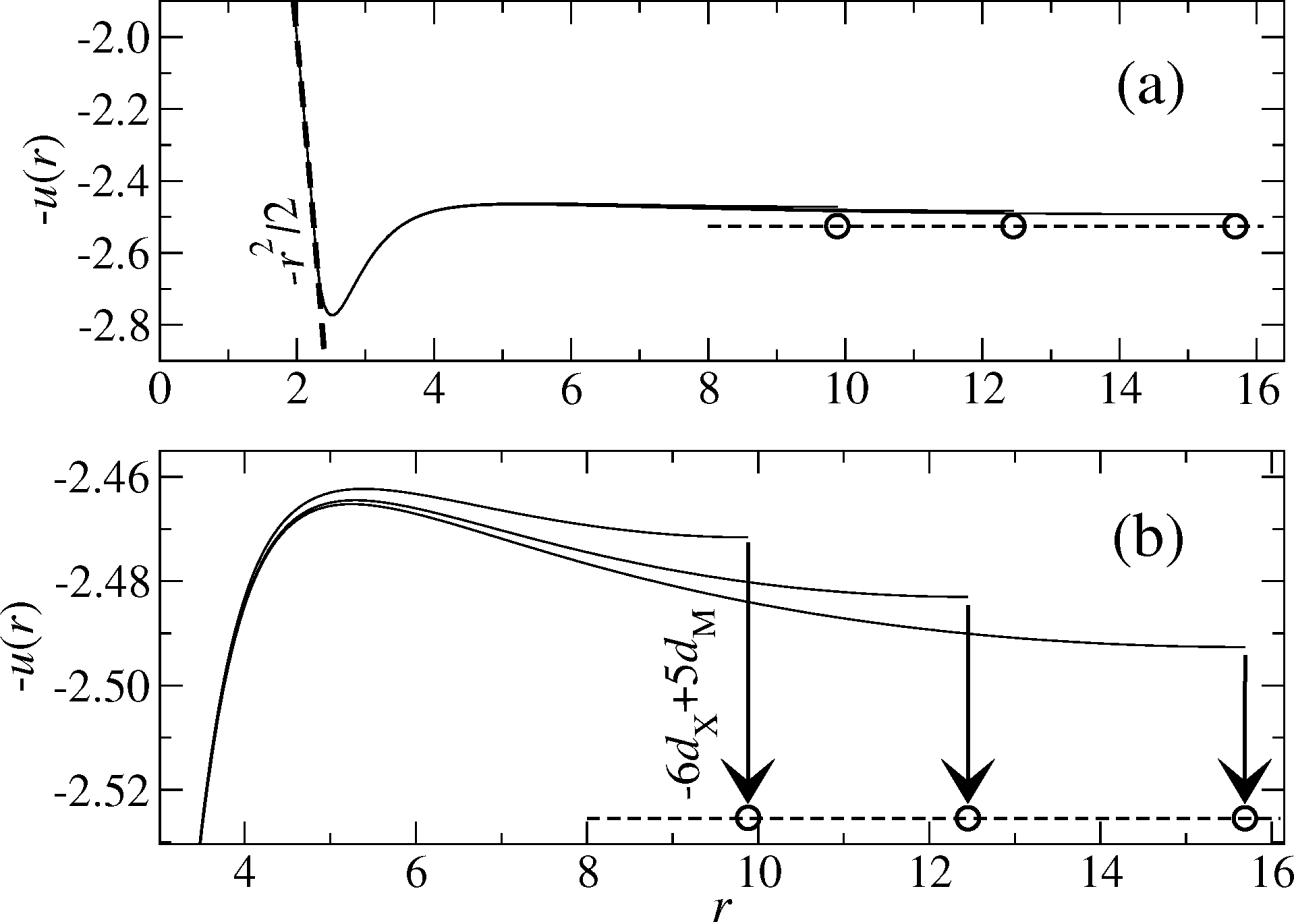
Negative elastic displacement −*u*(*r*) (as defined in Figure 1) of a tip with *R* = *E* = 1 pressed against a rigid substrate at a positive load of *F*_N_ = 1.5625 for a work of adhesion Δγ = 1 and a Tabor coefficient μ_T_ = 1 resulting in an exponential decay length of *z*_0_ = 1. In each case, the system is discretized into 512 × 512 grid points, but different sizes are used, i.e., *L* = *L*_0_, *L* = 2^1/3^*L*_0_, *L* = 2^2/3^*L*_0_ with *L*_0_ = 9.8825. Part (a) shows a larger domain including the shape of the tip in form of a dashed line. Part (b) shows a smaller domain and includes a higher-resolution estimate of the displacement at infinite radius *R* through the extrapolation 6*u*_X_ − 5*u*_M_.

[32]
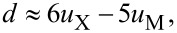


where X and M denote the (symmetry) points (*L*/2,*L*/2) and (*L*/2,0) relative to the position of the center of the tip. The same extrapolation scheme also appears to give quickly converging estimates for the normal displacement for adhesive contact, which is demonstrated in Figure 4.

It is worth discussing Figure 4. At the given load, the contact radius is *a*_c_ ≈ 2.30, while it would have been *a*_c_ ≈ 1.05 without adhesion. The displacement curve has a peak at *r* = 2.52 and adhesive effects remain non-negligible all the way up to *r* ≈ 4. At that distance the gap starts to be greater than 5*z*_0_, which means that the adhesive attraction is less than *e*^−5^ times the value in the contact and its immediate periphery. For distances exceeding *r* = 4, an infinitely large system would then show the displacement given in Equation 31. The periodic boundary conditions suppress this scaling rather strongly, yet, for radii as small as *r* = 10, accurate estimates for *d_∞_* can be achieved through Equation 32.

Simulations could be made more efficiently by exploiting the radial symmetry of the system. This would allow one to reduce sums over two indices (e.g., *q*_1_ and *q*_2_) to that over one index. However, less is gained than it first might seem. To get a good resolution of the contact area, the one-dimensional (1D) calculation require greater ratios for *a*_c_/*a* than two-dimensional setups. The reason is that the resolution of the contact area in 1D and in 2D both scale with 1/*N*, where *N* is the number of grid points in the contact. For example, when representing a contact in which for the given discretization 5*a* < *a*_c_ < 6*a* in a 2D system, then *a*_c_ is allowed to take the values 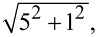 
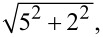
 
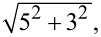
 and 
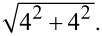
 The maximum distance between two radii thus is Δ*a*_max_ = 0.28 so that the resolution is Δ*a*_c_/*a* ≈ 5/0.28. To match this in a 1D model, one would need 18 grid points rather than five. Since we need to cover a (square) area of (2*a*_c_)^2^ in 2D, we thus have a computational overhead of a little more than a factor of 4 compared to 1D. However, the disadvantage of large 1D systems is that the number of iterations to find solutions can be much larger than in 2D. Depending on the nature of the solver, the number of iterations scales as a power law with linear system size. In the given case, where the effective stiffness scales proportional to wave vector *q*, one would expect a slowing down with 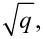 at least in simple gradient-based minimization such as steepest descent or molecular-dynamics. For this reason, no efforts were made to reduce the dimensionality, although this would have been legitimate for the given problem.

### Positive work of adhesion

This section analyzes how the employed models reproduce established results for adhesive single-asperity contacts in the limits of large and small Tabor coefficient. This includes an asymptotic analysis of Maugis’ solution of the Dugdale model, which in turn leads to modifications of the equations proposed by Carpick, Ogletree, and Salmeron [1]. The cross-over from JKR to DMT is investigated as well, in particular at zero load and near pull-off, allowing one to identify the model for the surface interaction that is most appropriate for the simulation of (adhesive) multi-asperity contacts.

#### Zero external load

An external load of *F*_N_ = 0 is addressed first. The motivation for studying this special case is that one can analyze relatively easily at what Tabor coefficients the DMT and JKR limits start to predict reasonably accurate values for the contact radii and displacements in our various models.

**Figure 5:**
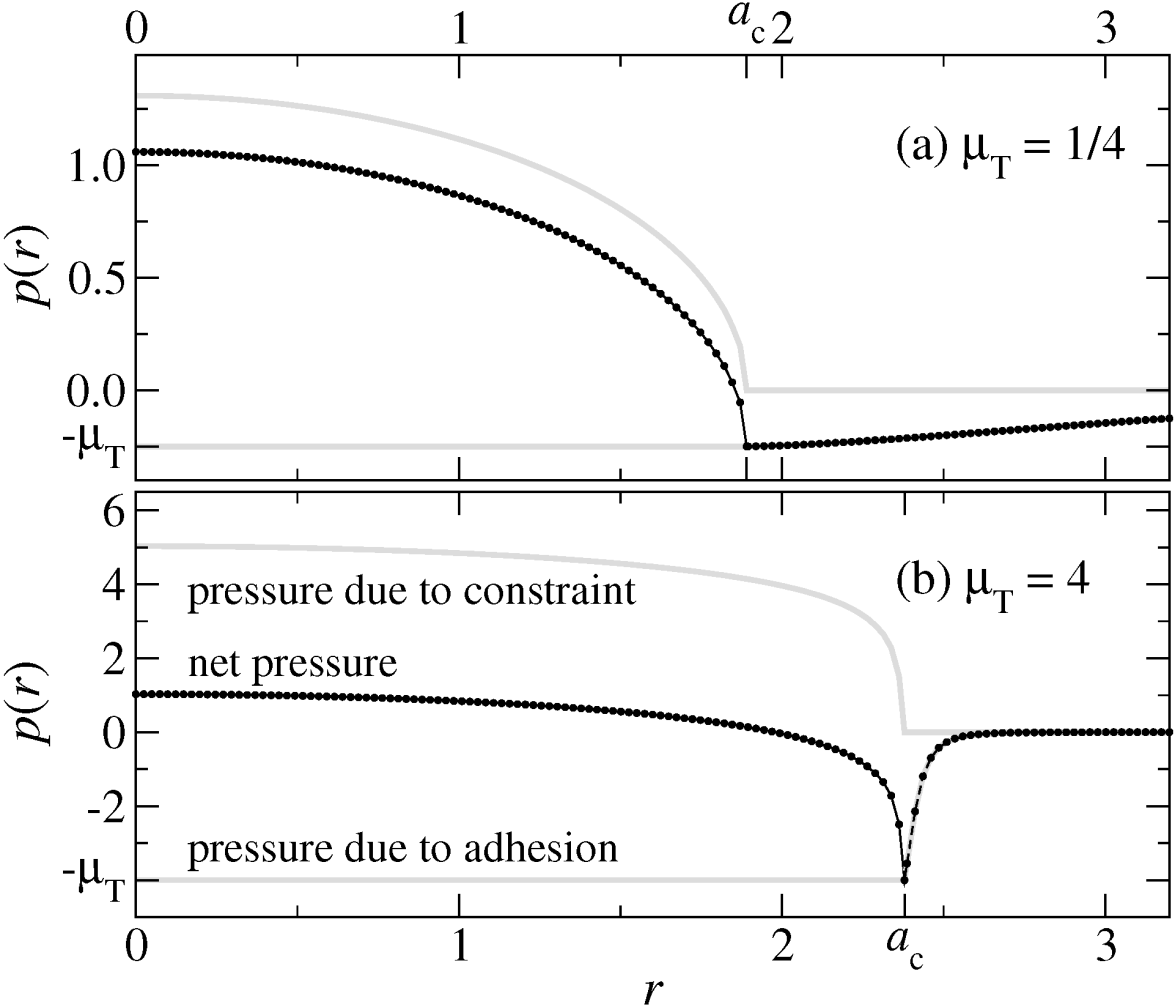
Interfacial pressure on a free, linearly-elastic half space resting at zero external load on a rigid, adhering, parabolic substrate for (a) μ_T_ = 1/4 and (b) μ_T_ = 4 in case of the exponential model. In each case, the upper and lower grey lines indicate, respectively, the pressure due to the constraint and that due to adhesion. Contact radii are indicated by *a*_c_. The net pressure is shown by a black line as well as by small circles representing the actual grid points. Units are chosen according to Equation 23–Equation 26.

We start our analysis with the pressure distribution of the exponential model, which is depicted in Figure 5 for μ_T_ = 1/4 and μ_T_ = 4. It behaves very similar to the MD model, which shall not be shown explicitly. As to be expected, the adhesive load is spread out for μ_T_ = 1/4 to radii clearly exceeding *a*_c_ (all the more as each radius *r* contributes with a weight proportional to *r*), while it is rather localized near *r* = *a*_c_ for μ_T_ = 4. It therefore seems legitimate to call the (net) pressure profile for μ_T_ = 1/4 DMT-like and JKR-like for μ_T_ = 4.

The adhesive pressure is calculated from the functional derivative *p*_adh_(*x,y*) = −δ*V*_fr_/δ*g*(*x,y*), where *V*_fr_ is defined in Equation 5. This can be evaluated to yield

[33]
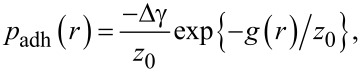


which becomes *p*_adh_(*r* < *a*_c_) = −Δγ/*z*_0_ within the true contact area. Using Equation 19, one obtains

[34]
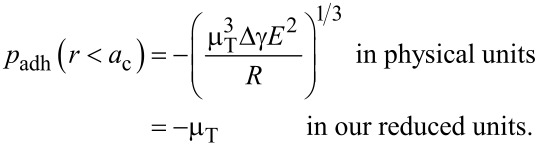


Stress or pressure originating from the constraint *g*(*x,y*) ≥ 0 is computed from the elastic Green’s functions.

**Figure 6:**
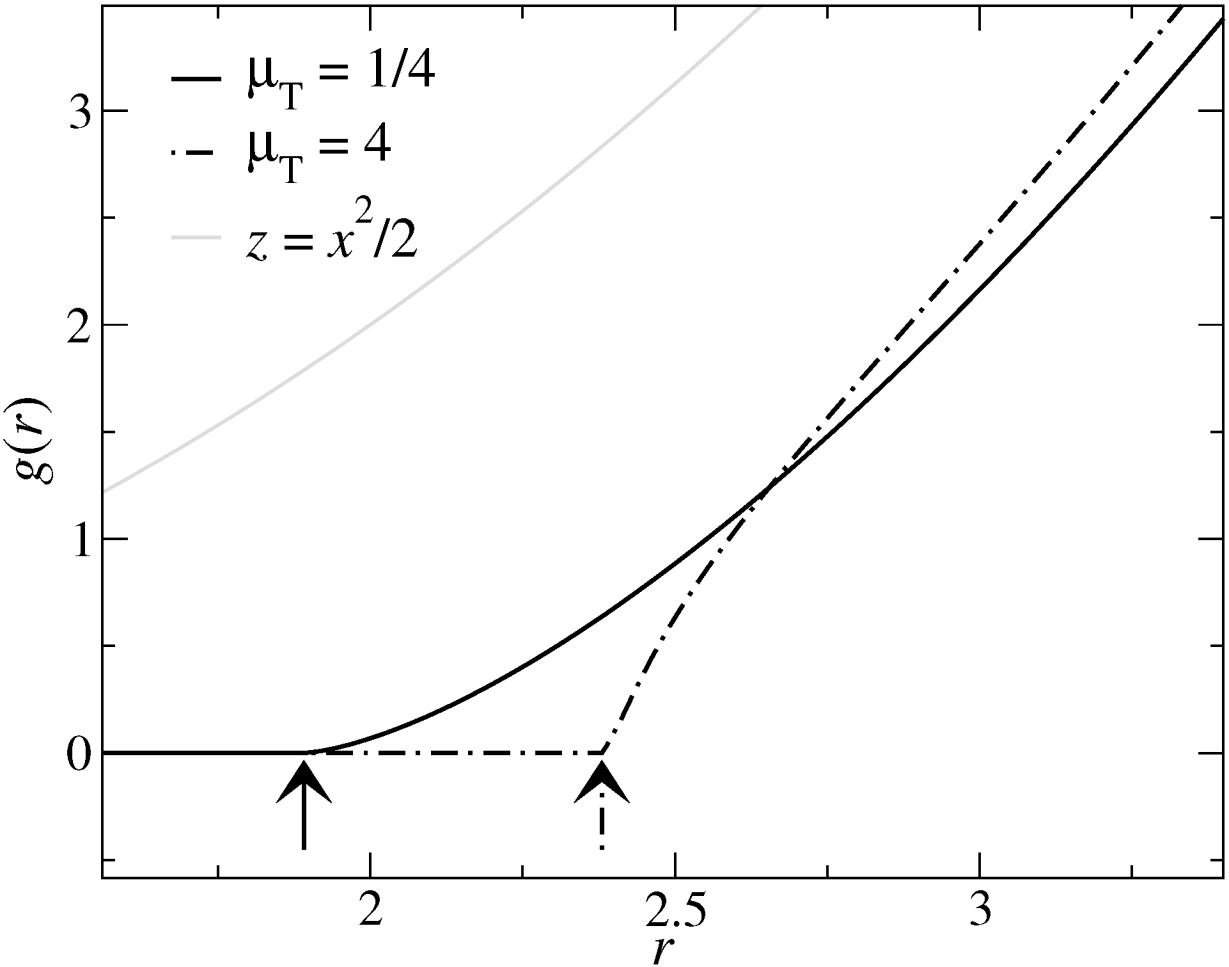
Gap between a rigid, adhesive, parabolic tip and a linearly-elastic half space for two different Tabor coefficients μ_T_ = 1/4 (solid black line) and μ_T_ = 4 (broken black line). The gap *z* = *x*^2^/2 of an undeformed half-space is shown in grey for comparison. Arrows indicate contact radii. Units are chosen according to Equation 23–Equation 26.

The well-known qualitative difference for the contact geometry of systems with large (μ_T_ = 4) and small (μ_T_ = 1/4) Tabor coefficient is borne out in the radial dependence of the gap *g*(*r*). Specifically, Figure 6 reveals that a small Tabor coefficient makes *g*(*r*) have a positive curvature at *r* ≥ *a*_c_, as in the DMT solution, while it has a negative curvature at *r* ≥ *a*_c_, indicative of an adhesive neck, for large μ_T_. Figure 6 also shows that the displacement (defined by the difference between the actual gap and the gap in an undeformed contact at *r* >> 1) is smaller for μ_T_ = 4 than for μ_T_ = 1/4, although the contact radius is larger for μ_T_ = 4 than for μ_T_ = 1/4.

**Figure 7:**
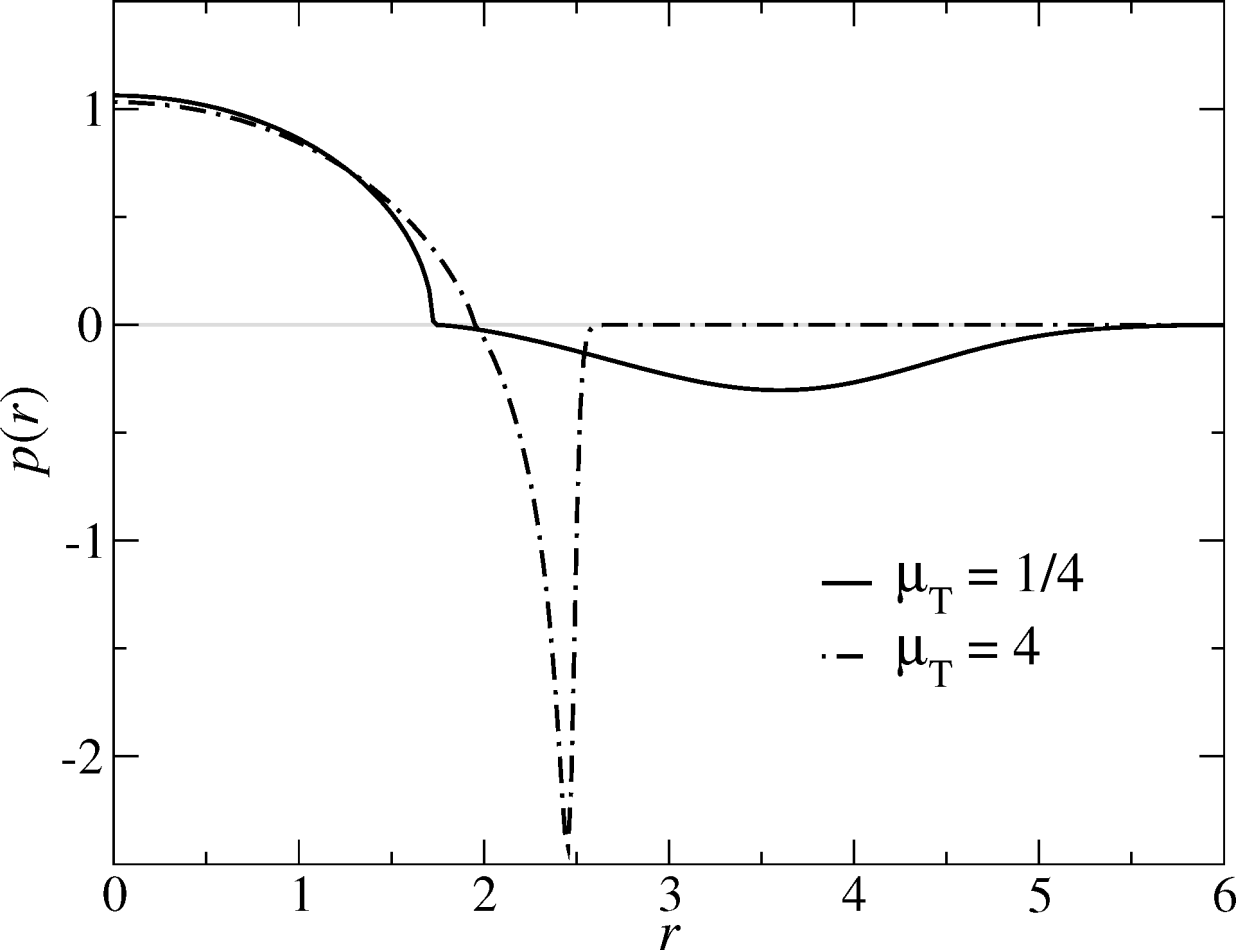
Pressure *p*(*r*) in the Gauss model at zero load for two different Tabor coefficients as a function of the in-plane distance *r* from the center of the contact. The contributions due to the constraint are positive, i.e., above the grey line, while the adhesive forces are negative.

The Gauss model behaves qualitatively different from the exponential model. First, there are no adhesive forces within the contact, but only outside of it, as shown in Figure 7. Second, at *r* = *a*_c_, the total pressure disappears in the Gauss model, while it remains finite in the exponential model. Third, the pressure due to the constraint has finite slope at *r* = 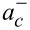 in the Gauss model, while the slope diverges in the exponential model (not shown explicitly). All these differences arise because the derivative of *v*_fr_(*g*/*z*_0_) remains finite (i.e., with positive sign) in the exponential model when the gap closes, while 
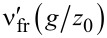
 is zero in the Gauss model.

**Figure 8:**
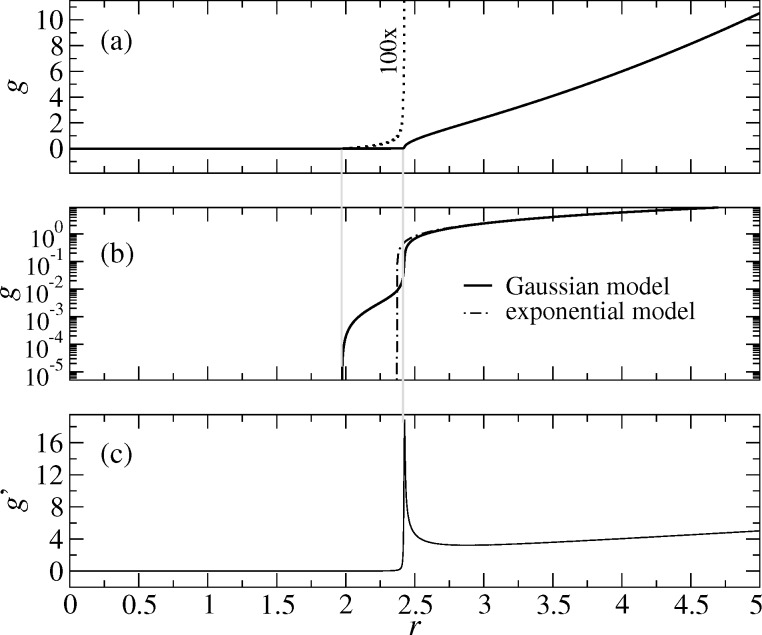
Gap *g*(*r*) in linear (a) and logarithmic (b) representation as well as (c) first derivative *g*′(*r*) for μ_T_ = 16 in the Gauss model (straight lines). A higher resolution representation of the gap is given in (a) with a dotted line. The exponential model is shown for comparison in (b). The two thin grey lines are drawn where the gap becomes zero (left line) and where the slope of *g*(*r*) takes its maximum value.

Another consequence of *p*_adh_(*g*/*z*_0_) having zero slope in the limit of *g* → 0^+^ is that the gap in the Gauss model closes continuously rather than with a discontinuity in its first derivative. This is shown in Figure 8, from where it becomes clear that it is difficult to define good measures for the contact radius. In a linear representation (at low resolution), it seems as if the contact closes with the typical adhesive neck, i.e., in part (a) of Figure 8 the gap appears to close at *r* ≈ 2.415. There, the slope of *g*(*r*) takes its maximum value, in a very similar fashion as in the JKR limit, or for the exponential model for the same value of μ_T_ = 16. However, when increasing the magnification, one can see that the contact closes only at *r* ≈ 1.97. Unfortunately, the radii where the gap closes to zero, and where *g*(*r*) has its maximum do not approach each other quickly when μ_T_ is increased. Instead, the value of *g* in the cross-over region in Figure 8b moves to smaller values as μ_T_ increases. Similar behavior is seen in the VDW model, which is not shown here explicitly.

**Figure 9:**
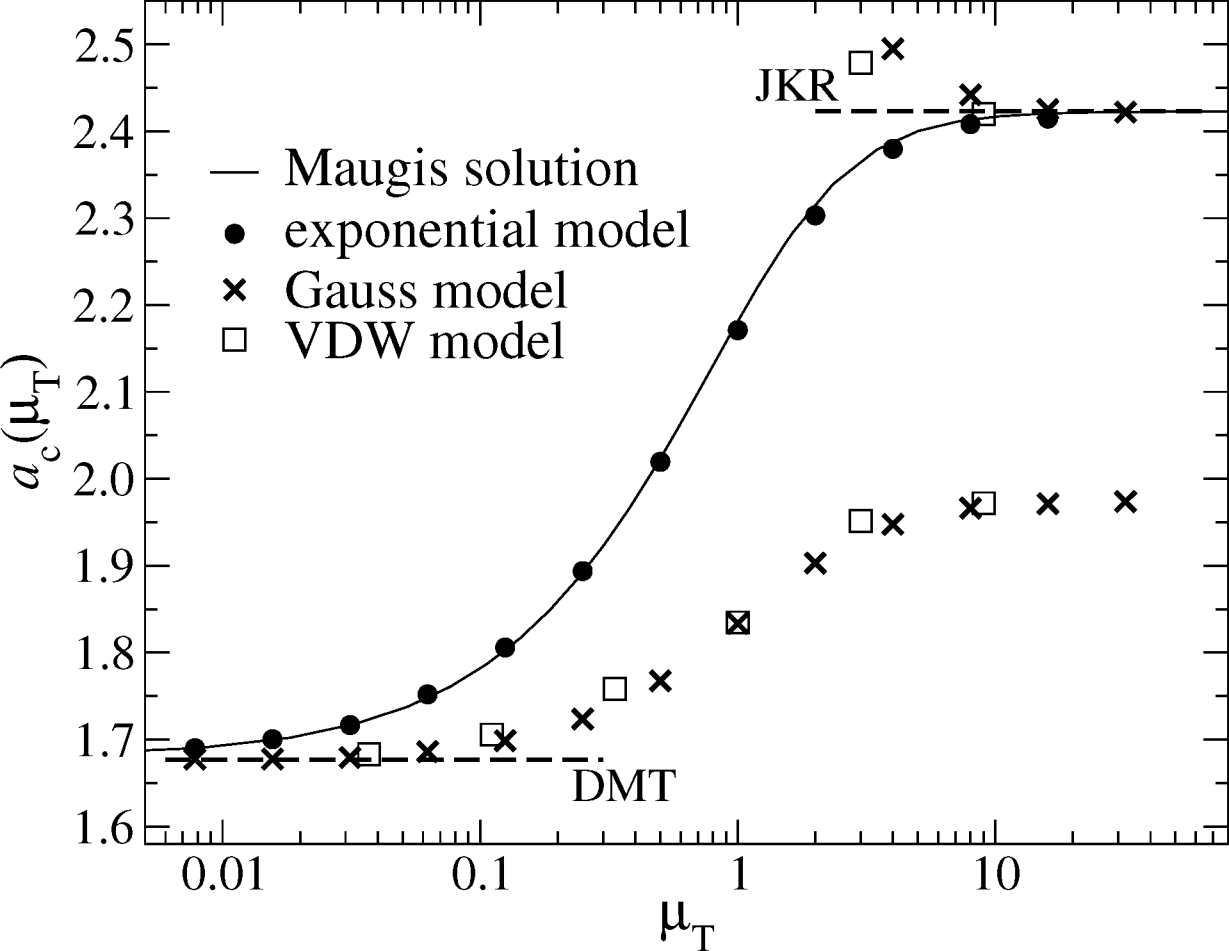
Contact radius *a*_c_ at zero external load as a function of the Tabor coefficient μ_T_ for different model interactions: exponential (full circles), Maugis Dugdale (straight lines), Gauss (crosses), and VDW (open squares). The upper and lower broken line denote the DMT and the JKR limits, respectively. In the Gauss and VDW models, two different definitions are used for *a*_c_: The upper symbols refer to the position where *g*′(*r*) reaches a local maximum, while the lower symbols indicate the points of first gap closure (*g* = 0).

Zero-load contact radii for different potentials are depicted in Figure 9 as function of the Tabor coefficient. In the exponential model, the contact radius approaches DMT and JKR limits in a very similar fashion as in the MD model. In a later section on the asymptotic analysis, I find that the MD corrections to the JKR limit are of order 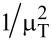 for large Tabor coefficients while those to the DMT limit are of order μ_T_ for small Tabor coefficients. The same scaling of the leading-order corrections is apparently borne out in the exponential model.

Models in which *v*_fr_(*g*/*z*_0_) has zero slope in the origin behave qualitatively different from the MD or the exponential model. They approach the DMT limit for the contact radius fairly quickly, i.e., roughly with 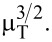 However, convergence to the JKR limit is poor. The latter can be improved by defining the contact line to be located where *g*′(*r*) takes its maximum value. Unfortunately, this definition cannot be universally applied, i.e., only when μ_T_ is sufficiently large to allow for an adhesive neck to be formed, see also Figure 8. Moreover, in the context of randomly rough surfaces with complicated contact geometries, this last definition of contact would not be practicable.

**Figure 10:**
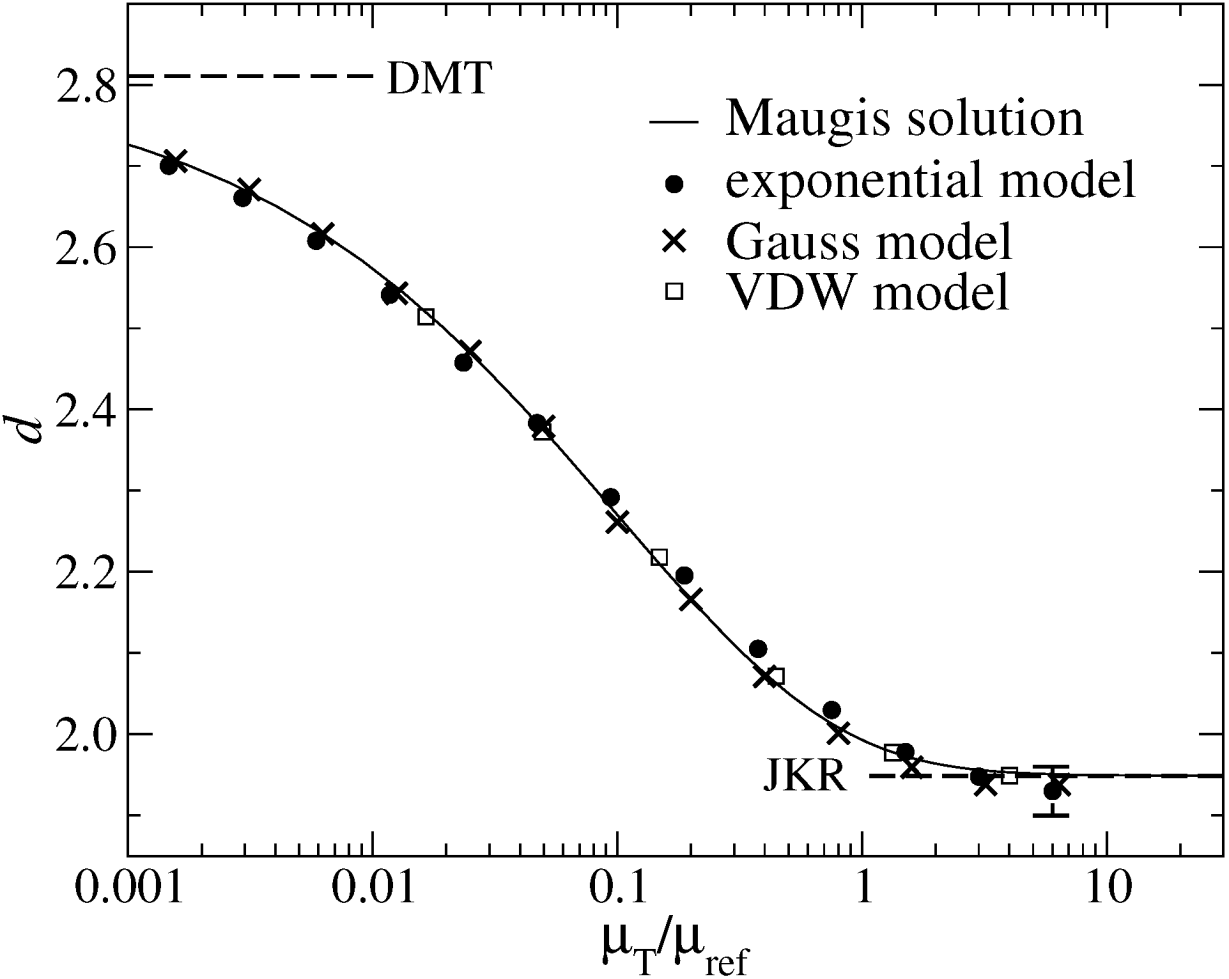
Normal displacement *d* at zero external load as a function of the Tabor coefficient μ_T_ for different model interactions: exponential (full circles), Maugis Dugdale (straight lines), Gauss (crosses), and VDW (open squares). The Tabor coefficient was normalized so that all curves superimpose at the “half-way” distance *d* = (*d*_JKR_ + *d*_DMT_)/2. The upper and lower broken line denote the DMT and the JKR limits, respectively. Numerical uncertainties arise in the limit of large Tabor coefficients, as indicated by the error bar.

Unlike the contact radius, the normal displacement *d* does not suffer from any difficulties to be properly defined. In principle, this could enable one to ascertain *v*_fr_(*g*) from displacement measurements without much ambiguity. However, Figure 10 reveals that the functional form of *d*(μ_T_) is relatively insensitive to the details of the finite-range interaction, at least, as long as we allow for a redefinition of the Tabor coefficient, such that all curves superimpose at the distance half way between the JKR and the DMT limit. This is in agreement with a work by Tvergaard and Hutchinson [18] who found that Δγ and the peak stress (which one may losely associate with Δγ/*z*_0_) are the basic parameters for mode I fracture.

Before proceeding to the case of finite load, I wish to comment on the relatively large numerical (GFMD) uncertainties for the displacement at large Tabor coefficients. They stem predominantly from the difficulty to apply the finite-size extrapolation formula, Equation 32, to gaps having adhesive necks. This problem would not be present in large-scale simulations of multi-asperity interfaces, because system sizes would automatically be much larger than local contact radii. One may conclude that the use of the exponential model for the study of adhesive multi-asperity contacts appears to be appropriate. The MD model could be used as well, in principle, however, it might induce undesired numerical instabilities due to the discontinuity of *v*′(*g*/*z*_0_) at *g* = *z*_0_. The Gauss model can only be taken when the property of interest is related to the gap but not for the calculation of contact area. If one wanted to simulate van der Waals attraction at large μ_T_, one might want to replace the VDW model in Equation 12 with 1/(1 + *g*/*z*_0_)^2^. This dependence makes it possible to determine the contact area meaningfully in the realm of continuum mechanics while using reasonable approximations for van der Waals interactions at large distance.

#### Finite external load

**Figure 11:**
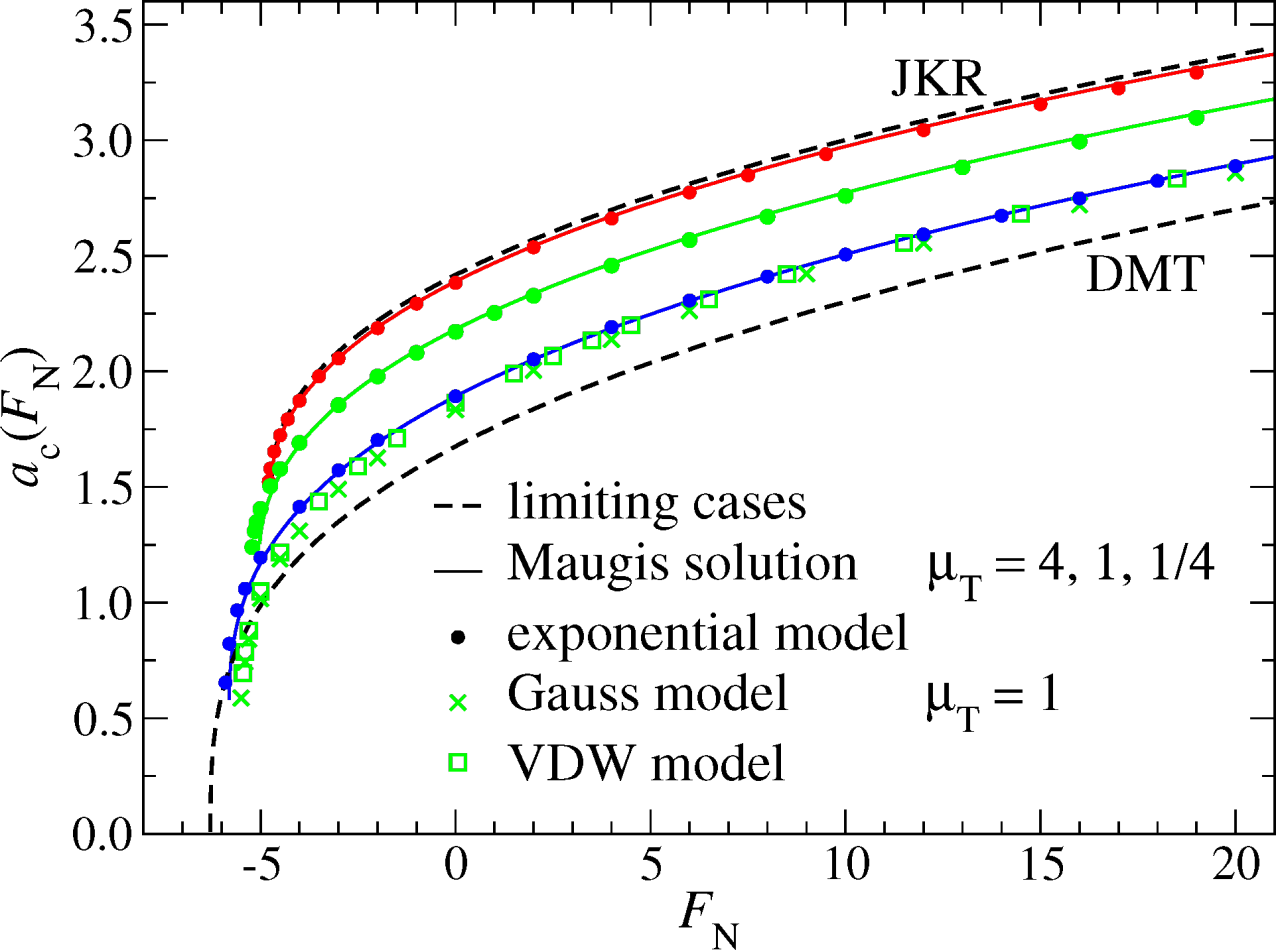
Contact radius *a*_c_ as a function of load *F*_N_ for the exponential and the MD model using different Tabor coefficients, ranging from μ_T_ → ∞ (JKR, top) to μ_T_ = 0 (DMT, bottom). For the Gauss and VDW models, only μ_T_ = 1 is shown. Their *a*_c_(*F*_N_) curve is similar to that of the Maugis and the exponential model for μ_T_ = 1/4. Color coding: μ_T_ = 4 (red), μ_T_ = 1 (green), and μ_T_ = 1/4 (blue).

In most experiments, the Tabor coefficient is kept constant and the normal load is changed. As a result, one obtains the normal displacement *d*(*F*_N_) as a function of the normal load *F*_N_. In some cases, i.e., for sufficiently large contact radii, an estimate of the contact radius, *a*_c_(*F*_N_), can be obtained as well. One might be tempted to believe that knowing such curves allows one to deduce the surface forces. Here, I want to investigate to what degree such an inversion is possible by studying the sensitivity of the functions *d*(*F*_N_) and *a*_c_(*F*_N_) to the functional form of the surface interactions. Figure 11 shows the contact radius as a function of the normal load. One can see that the exponential model and the MD model agree very closely, that is, curves almost superimpose for a given Tabor coefficient. This makes it essentially impossible to discriminate between these two forms of interaction experimentally. Likewise, the Gauss and VDW models also coincide for the same Tabor coefficient despite their significant differences at large gaps. Interestingly, the μ_T_ = 1 curve for VDW and Gauss (both having finite slope potentials at *g* = 0) is akin of the μ_T_ = 1/4 curves for the MD and the exponential model (both having zero-slope potentials at *g* = 0). This confirms the trend reflected in Figure 9: Surface potentials with zero slope at *g* = 0 make the results move toward the DMT limit.

**Figure 12:**
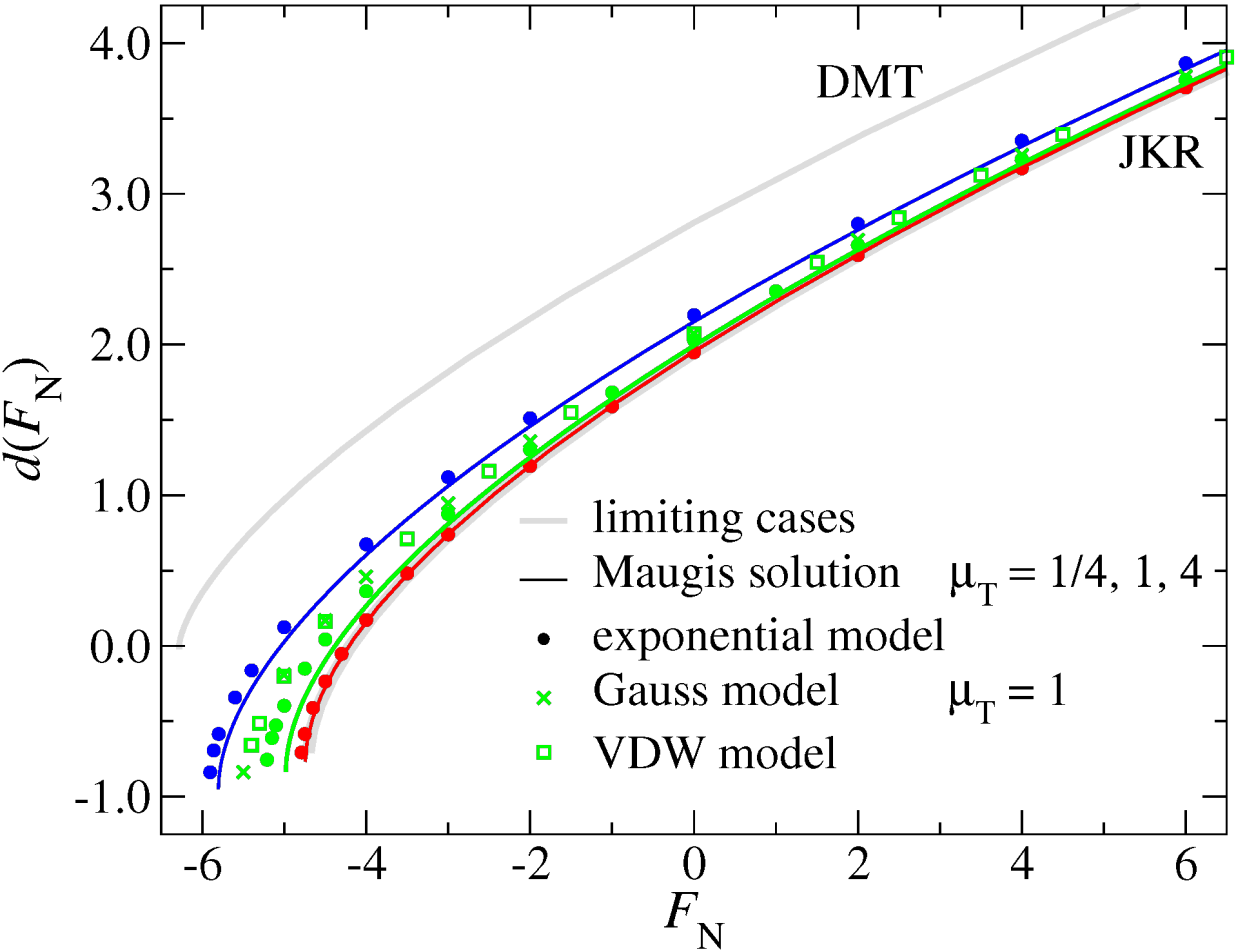
Normal displacement *d* as a function of load *F*_L_ for the exponential and the MD model using different Tabor coefficients, ranging from μ_T_ = 0 (DMT, top) to μ_T_ → ∞ (JKR, bottom). For the Gauss and the exponential model, data is only shown for μ_T_ = 1. Color coding: μ_T_ = 4 (red), μ_T_ = 1 (green), and μ_T_ = 1/4 (blue).

In contrast to the *a*_c_(*F*_N_) dependence, the normal displacement curve *d*(*F*_N_) predominantly depends on the Tabor coefficient. Now, all μ_T_ = 1 curves resemble each other closely, independent of the slope of the surface potential at zero gap. As for the normal displacement, all curves are reasonably close to the JKR limit. Even the μ_T_ = 1/4 curve lies closer to the JKR than to the DMT line. This is consistent with the results shown in Figure 10, which show that the DMT limit for *d*(*F*_N_) is only reached at extremely small Tabor coefficients. Figure 12 reveals that it is possible to adjust the free parameters of the MD model to fit *d*(*F*_N_) curves for a broad variety of surface interactions. However, one should abstain from deducing contact areas based on such fits, as this can result in non-negligible errors. For example, if we only knew the contact area from Maugis’ solution, we would be ill-advised to conclude from Figure 12 that the contact area for the μ_T_ = 1 Gauss model should lie roughly half way between those of the μ_T_ = 1 and μ_T_ = 4.

#### Comparison to other models and asymptotic analysis

Maugis proposed an analytical solution for the relation between contact radius *a*_c_ and normal load *F*_N_ in the Dugdale model. It requires the elimination of an auxiliary variable, *m*, through the self-consistent solution of two coupled non-linear equations. Once *a*_c_ and *m* are found, the displacement *d* can be readily calculated as well. Using tildes to indicate variables in Maugis’ unit system, the relevant equations read:

[35]
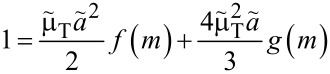


[36]
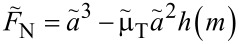


[37]
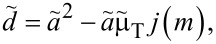


where the functions *f*(*m*), *g*(*m*), *h*(*m*), and *j*(*m*) are defined as

[38]
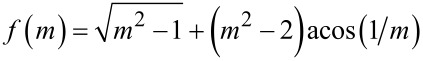


[39]
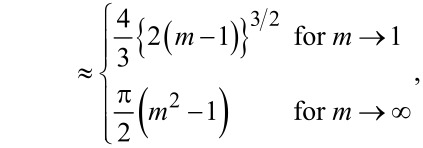


[40]
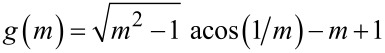


[41]
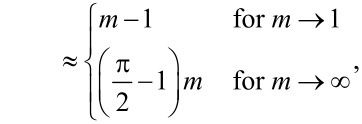


[42]
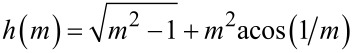


[43]
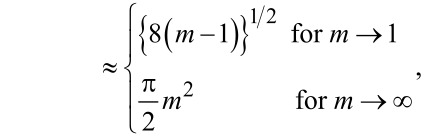


and

[44]
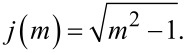


In each but one (straightforward) case, behavior of the functions for *m* approaching unity or infinity has been included. They become useful in the limit of large and small Tabor coefficients, respectively.

Conversion back to our unit system can be done using:

[29]
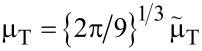


[45]
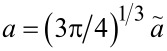


[46]
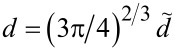


[30]
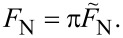


To overcome the need of having to find the self-consistent solution to Maugis’ equations, Carpick, Ogletree, and Salmeron (COS) [1] proposed a simple and thus elegant analytical formula for the *a*_c_(*F*_N_) dependence

[47]
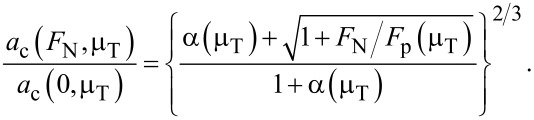


Schwarz [2] later recognized that the COS description is exact – given proper parameterization – if the interaction between the surfaces results from the superposition of an infinitesimally short-ranged and an infinitely long-ranged contribution. However, in the given context of one intermediate-range potential, I will treat the COS equation as a guessed approximation containing the correct functional form in the limits of large and small μ_T_.

The primary COS equation (Equation 47) is designed such that the contact radius at zero load *a*_c_(0,μ_T_) as well as the pull-off force *F*_p_(μ_T_) can be reproduced exactly. However, approximations to their dependence on μ_T_ had been provided as well, because no closed-form expression are available. A free parameter remains, α(μ_T_), which can be used to minimize deviations from the exact solution. At large loads, one recovers the well-known *a*_c_ 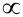 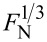 scaling, however, not necessarily with the correct prefactor. Another property of the COS approximation is that it does not necessarily contain the correct value of the contact radius at pull-off. Thus, despite predicting the contact radius fairly well, the COS contact radius is not exact in the limit of very large and very small (i.e., pull-off) normal loads. These deficiencies can be improved when parameterizing the COS equation in a slightly different fashion, e.g.,

[48]
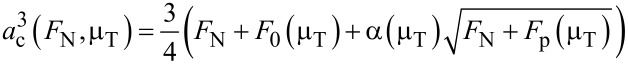


with

[49]
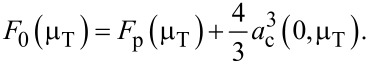


This set of equation ensures that *a*_c_ converges to the exact value when *F*_N_ → ∞ and *F*_N_ → −*F*_p_. The parameter α(μ_T_) can then be adjusted to either yield the correct zero-load contact radius, or to minimize the deviation between approximation and the exact Maugis solution by some other mean. Note that the factors 3/4 in Equation 48 and 4/3 in Equation 49 have to be replaced by unity when working with Maugis’ unit system.

**Figure 13:**
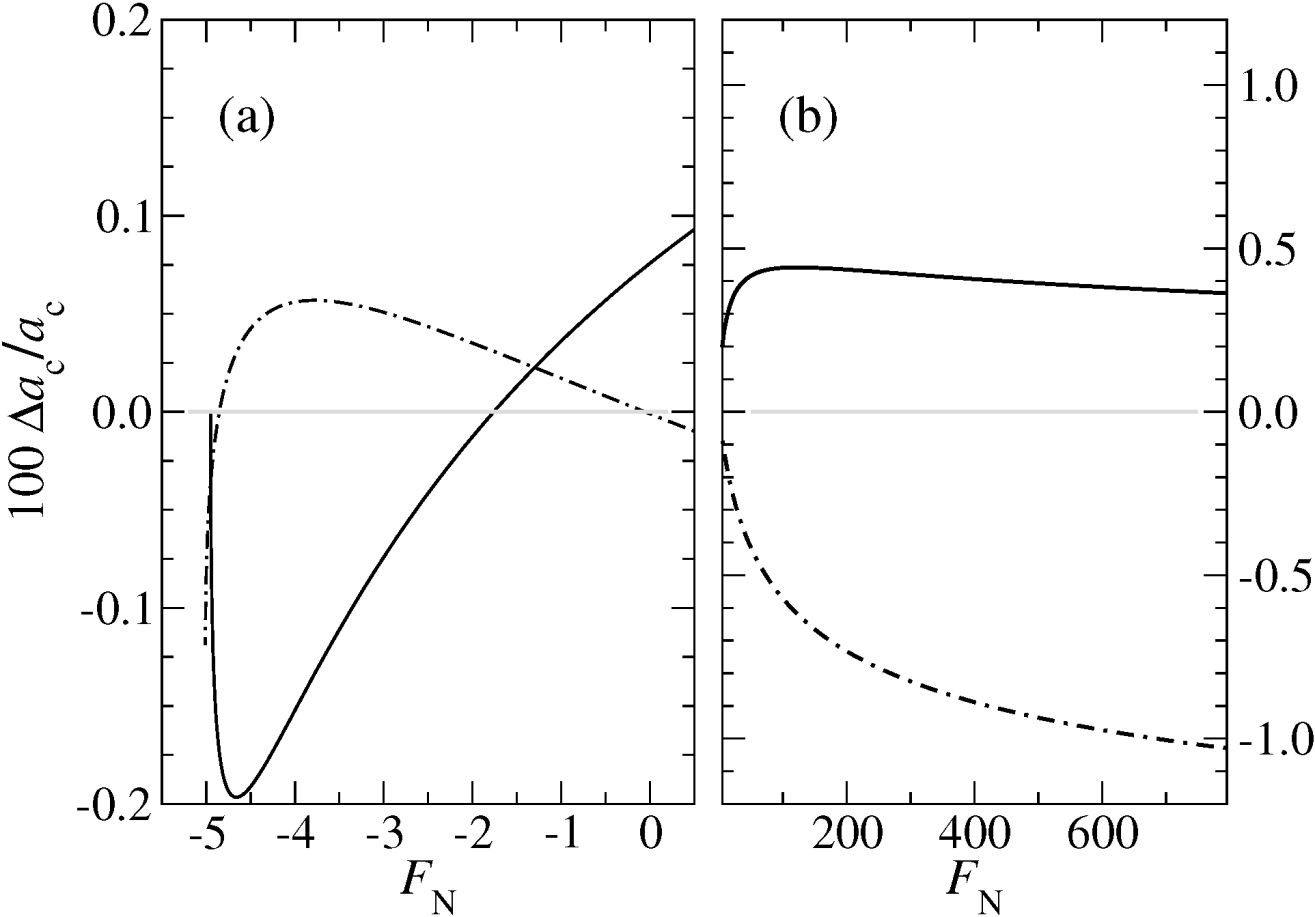
Relative errors in per cent for the contact radius *a*_c_ for μ_T_ = 1 at (a) negative and (b) positive load. The full line indicates the error when using Equation 48, while the dot-dashed line is based on Equation 47. In both cases, the parameter α(μ_T_ ) was adjusted to minimize the deviation from Maugis solution in the domain −*F*_p_ < *F*_N_ < 2*F*_p_.

I wish to note that including the correct asymptotics in the *a*_c_(*F*_N_) expression does not necessarily improve the fits in the range from slightly above the pull-off force at negative loads to several times the absolute pull-off force. This is demonstrated in Figure 13. Moreover, convergence to the correct *a*_c_(*F*_N_) dependence at large loads is rather slow even when using Equation 49.

It is also possible to constrain the COS relation for the contact radius such that it contains the correct pull-off force and contact radius as well the correct zero-load radius. In either case, relative errors are small, i.e., ≤1% even for μ_T_ ≈ 1, where one is relatively far away from both the DMT and the JKR limit.

**Zero load:** The asymptotic analysis is readily done for zero loads, because the variable *m* can be directly eliminated in that case. As a result, one obtains

[50]
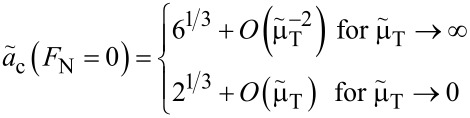


and

[51]
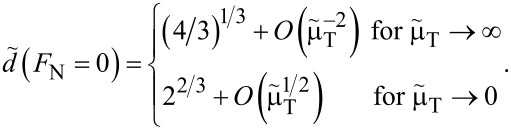


From the last two equations, one can see – as in Figure 9 and Figure 10 – that the JKR limit is quickly reached as the Tabor coefficient increases. However, convergence to the DMT limit with decreasing 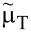 is rather slow. It is particularly slow for the normal displacement. E.g., to have a maximum error in *a*_c_(*F*_N_ = 0) and *d*(*F*_N_ = 0) that is of order 1% with respect to a desired limit, it is sufficient to work with 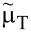 ≈ 10 for JKR, but one needs 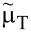 ≈ 10^−4^ to approach the DMT limit with similar accuracy. The latter is not problematic for the simulation of multi-asperity contacts, as the system is large by default. However, for single-asperity contacts, large deviations from μ_T_ = 1 (on a logarithmic scale) are difficult to handle in single-asperity contact simulations for reasons discussed in the numerical-analysis section.

Knowing the asymptotic behavior of 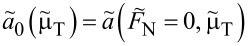 and 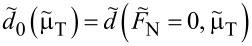 with respect to 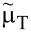 allows one to incorporate it in empirical equations for these two quantities. The following equations are found to achieve this and to provide excellent approximations to Maugis’ solutions:

[52]
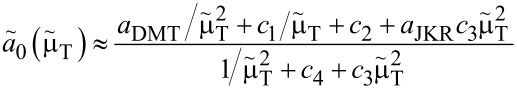


[53]
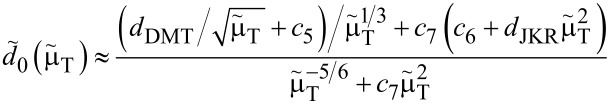


**Figure 14:**
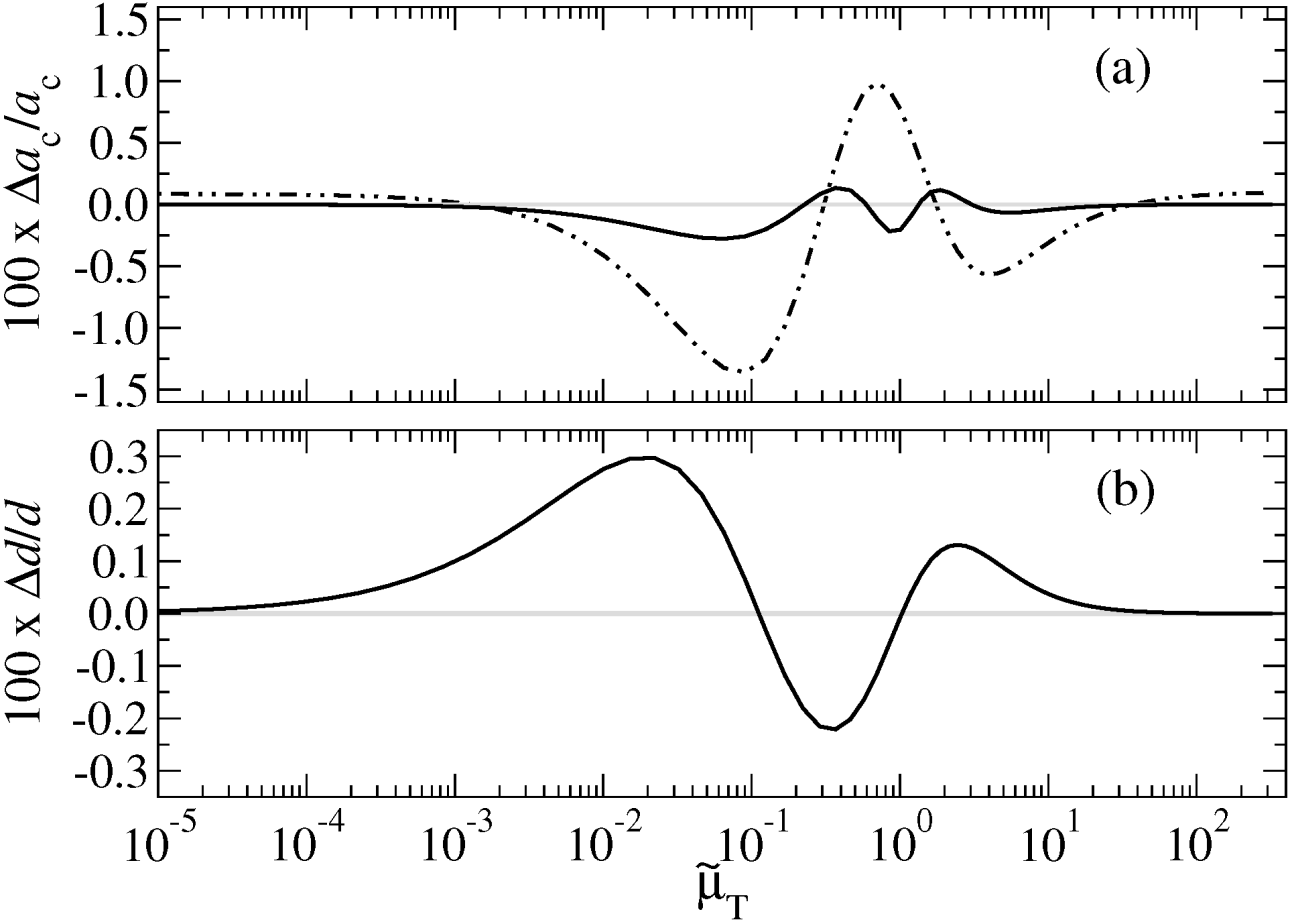
Relative errors in per cent for (a) contact radius and (b) normal displacement at zero normal load. Full lines refer to a fit based on Equation 52 and Equation 53 containing the correct asymptotic limits. The dotted line reflects an empirical fit based on the COS equations.

Two coefficients in each of the last two equations (*c*_1_, *c*_2_ and *c*_5_, *c*_6_) can be constrained to reproduce the correct asymptotics (and thus be obtained analytically). Two fit parameters remain for contact radius and one for the displacement. The relative errors from the pertinent fits are shown in Figure 14. Compared to an already quite accurate empirical relation proposed by Carpick et al. for 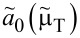, see Eq. (12b) in [1], the new Equation 52 and Equation 53 contain the correct asymptotics and reduces the maximum relative error from 1.5% to 0.3%. The data shown in Figure 14 were obtained with the following numerical values: *c*_1_ = 4/5, *c*_2_ = −1.285, *c*_3_ = 4/5, *c*_4_ = −0.435, *c*_5_ = −3/2, *c*_6_ = 0.1845, and *c*_7_ = 6.71.

**Figure 15:**
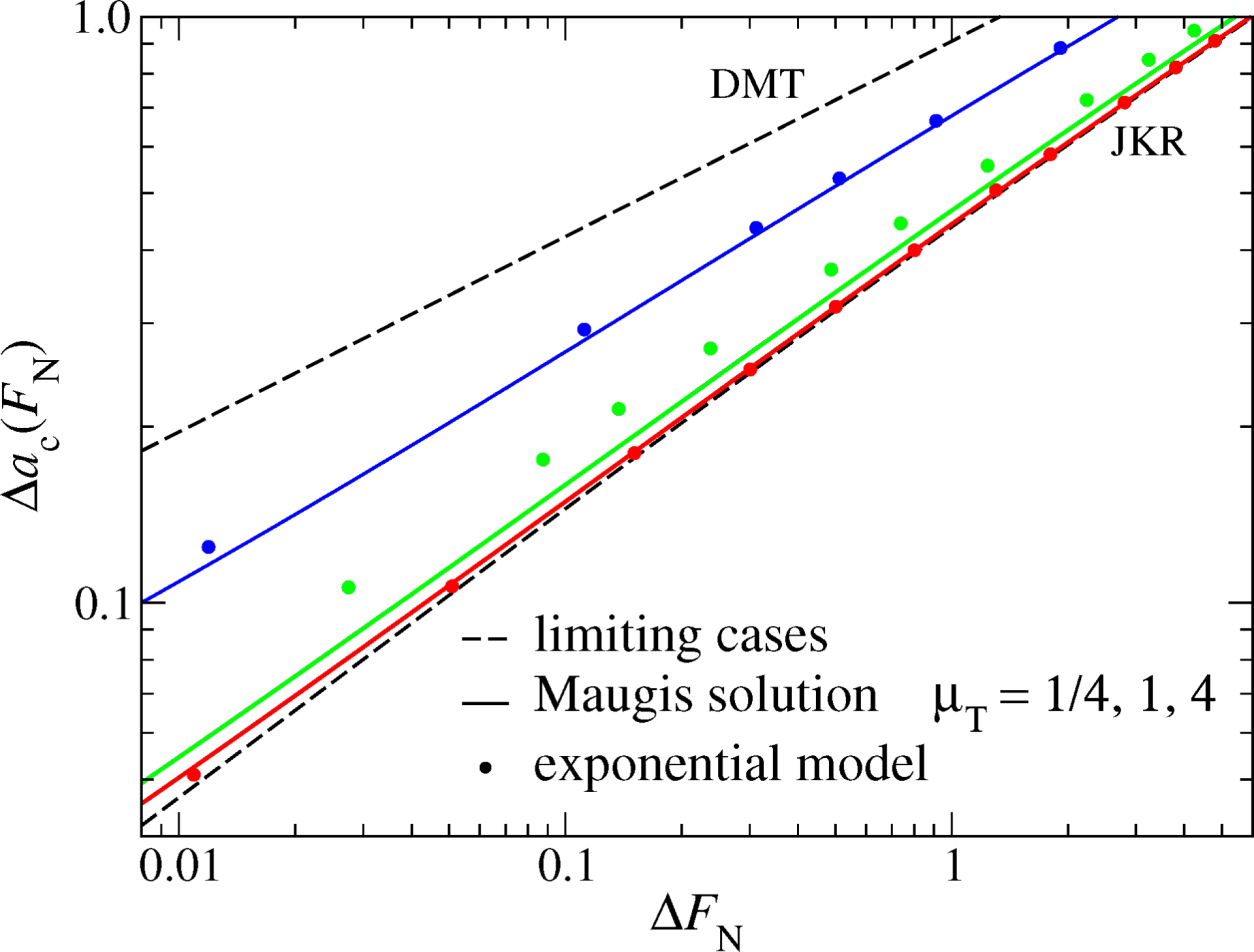
Excess contact radius Δ*a*_c_ = *a*_c_ − *a*_p_ as a function of the excess load Δ*F*_N_ = *F*_N_ + *F*_p_ for different values of the Tabor coefficient ranging from μ_T_ = 0 (DMT, top) to μ_T_ → ∞ (JKR, bottom). Here *a*_c_ and *a*_p_ denote the contact radius at an arbitrary load *F*_N_ and the pull-off load *F*_p_, respectively. Deviations between the Maugis solution and the exponential model are particularly obvious for the μ_T_ = 1 data set. Color coding: μ_T_ = 4 (red), μ_T_ = 1 (green), and μ_T_ = 1/4 (blue).

**Asymptotic behavior near pull off:** The structure of the COS approximation, Equation 47, and its modified form, Equation 48, indicates that the critical behavior near pull off satisfies 
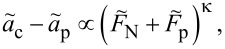
 where κ must be either κ = 1/3 as in the DMT limit, or κ = 1/2 as in the JKR limit. However, nothing in the self-consistent solution of Maugis indicates that the exponent κ changes discontinuously from one value to the next as the Tabor coefficient reaches or passes through a critical value. In fact, representing the data from Figure 11 in terms of Δ*a*_c_(Δ*F*_N_), as done in Figure 15, shows that κ changes continuously from 1/3 to 1/2 as μ_T_ increases from 0 to infinity.

An analysis for the normal displacement, similar to the one presented in Figure 15 but not shown explicitly here, exhibits a similar trend. The exponent describing Δ*d* = *d* − *d*_p_ as a function of Δ*F*_N_ = *F*_N_ + *F*_p_ crosses over continuously from the DMT to the JKR limit as μ_T_ increases. However, there is not a one-to-one relation between μ_T_ and κ. In particular the data sets for μ_T_ = 1 show relatively large differences between the exponent in the MD model (κ ≈ 0.469) and the exponential model (κ ≈ 0.435).

The insights obtained from Figure 15 can be used, in principle, to further modify the COS approximations, e.g., by replacing the square-root in Equation 48 by some other power or likewise by changing the square-root and the exponent 2/3 on the r.h.s. of Equation 47 in an appropriate fashion. When doing so, the modified version of Equation 48 does not only converge to the correct value at pull-off. It can also be parameterized to yield the correct asymptotics near pull-off. This results in a further reduction of the mean or overall error by a little more than a factor of two with respect to those shown in Figure 13, however, at the expense of one additional fit variable. Since the main new aspect of this study is concerned with negative work of adhesion and, moreover, both original and modified COS equations are already quite accurate, a more detailed analysis of the adhesive single-asperity contact is not pursued in this work.

### Negative work of adhesion

For repulsive contacts, Δγ < 0, there is obviously no finite contact radius at zero normal load *F*_N_ = 0^+^. The repelled rigid tip simply “hovers” at (infinitely) large distance over an undeformed elastic manifold. This is why it is not possible in this case to conduct a zero-load analysis similar to that presented for adhesive contacts. Since Maugis’ solution has not yet been extended to repulsive contacts, we are not in a position to compare our data to analytical solutions for negative Δγ. One of the consequences is that the asymptotic analysis must be based on GFMD data, except for μ_T_ → 0, for which normal forces couple predominantly to long-wavelength modes so that the Hertz-plus-offset approximation (DMT) should be accurate. Given the close similarity between the exponential and the Maugis–Dugdale model as well as that between the Gauss and the VDW model seen in the last section, the attention is restricted to one potential in each class, i.e., the exponential and the Gauss model.

**Figure 16:**
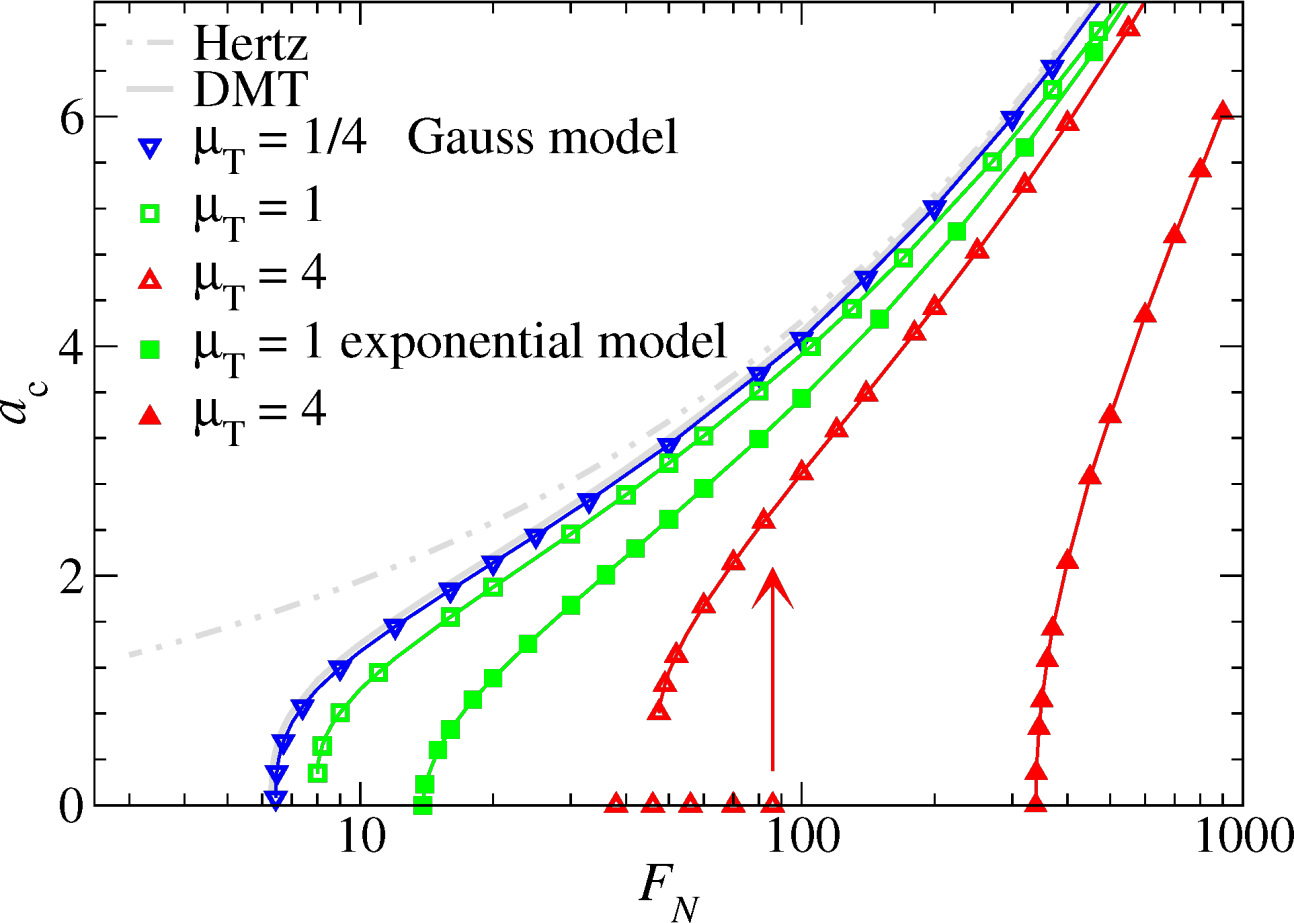
Contact radius *a*_c_ as a function of normal force *F*_N_ for the exponential (full symbols) and the Gauss (open symbols) model. Lines connect data points (not all shown explicitly). In the case of μ_T_ = 4 (Gauss model), an arrow indicates where the *a*_c_ = 0 solution becomes unstable for increasing *F*_N_. Color coding: μ_T_ = 4 (red), μ_T_ = 1 (green), and μ_T_ = 1/4 (blue).

We start our analysis with the contact radius dependence on load. In analogy to the context of wetting fluids, one may call the force at which a finite value of *a*_c_ becomes unstable upon lowering the load the spontaneous wetting force *F*_sw_. The force above which *a*_c_ can no longer be zero is called the squeeze-out force *F*_sq_. If the transition from contact to non-contact is continuous *F*_sw_ = *F*_sq_, otherwise *F*_sw_ < *F*_sq_. Results are shown in Figure 16.

As is the case for attractive interactions, the contact radius at small loads can be sensitive to both the Tabor coefficient and the choice of the potential. Specifically, the exponential model always shows a continuous transition from finite to zero contact radius (at least for the values of μ_T_ investigated here), while the Gauss model has either a continuous transition below a critical Tabor coefficient 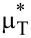 ≤ 1 or a discontinuous transition for μ_T_ > 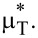 The discontinuity of the contact radius for Gauss potentials and sufficiently large Tabor coefficients implies that two solutions may coexist, i.e., one where the two surfaces are separated and one where they touch. However, once *F*_N_ exceeds a second threshold force *F*_sq_(μ_T_), i.e., the squeeze-out force, only one solution survives, that is, the one with finite contact radius. This can be seen in analogy to adhesive contacts with μ_T_ > 0, where two solutions coexist in a finite interval of forces −*F*_p_ ≤ *F*_N_ ≤ 0.

As for the *a*_c_(*F*_N_) relation near pull-off in the case of positive work of adhesion, the excess contact radius, *a*_c_ − *a*_sw_, depends as a power law on the excess force, *F*_N_ − *F*_sw_, for *F*_N_ ≥ *F*_sw_:

[54]
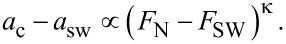


**Figure 17:**
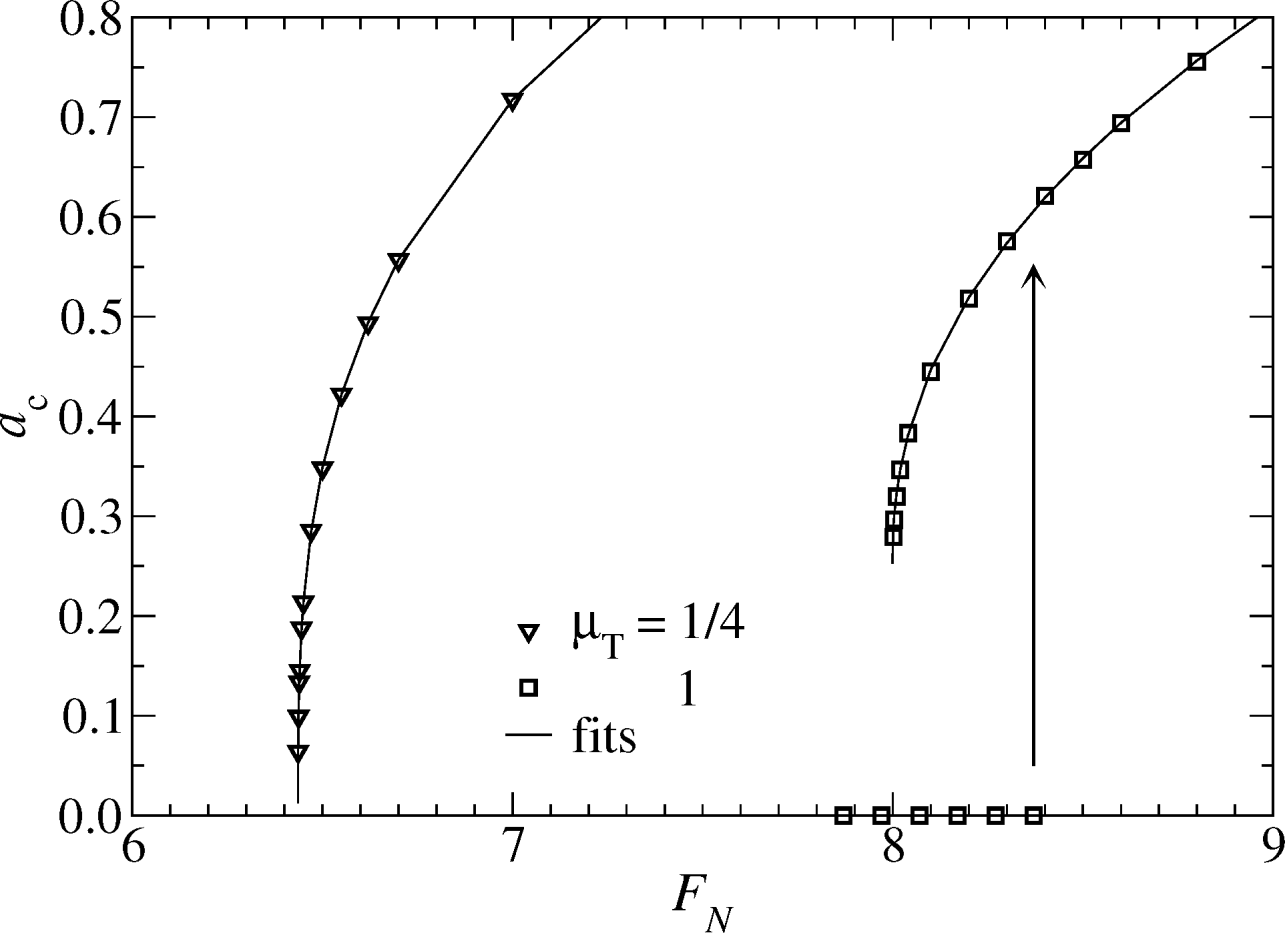
Contact radius *a*_c_ as a function of normal force *F*_N_ in the vicinity of the spontaneous wetting force *F*_sw_. Symbols reflect numerical results while lines are fits according to Equation 54. They terminate at *a*_c_(*F*_sw_). In the case of μ_T_ = 1, an arrow indicates where the *a*_c_ = 0 solution becomes unstable with increasing *F*_N_.

Fits to the *a*_c_(*F*_N_) relation are shown exemplarily for two values of μ_T_ in Figure 17. Details about the fits to the presented as well as additional data are summarized in Table 1. As for attractive contacts, it is found that κ changes continuously from κ(μ_T_ → ∞) = 1/2 to κ(μ_T_ → 0) = 1/3. For small μ_T_ , Hertz-plus-offset behavior is reached as evidenced by the observation that *c* and *F*_sw_ approach (3/4)^1/3^ and 2π, respectively. However, *F*_sw_ as well as *F*_sq_ quickly increase with μ_T_ for μ_T_ ≥ 1. This latter behavior is different from that of the pull-off *F*_p_ force for attractive surfaces, which only varies between 1.5π and 2π in the present unit system. Since the increase of both *F*_sw_ and *F*_sq_ is much faster in the exponential model than in the Gauss model, one can conclude that the exponential model converges more quickly to the continuum model than the Gauss model.

**Table 1:** Results of fits to the data shown in Figure 16. The last digit may not be significant.

model	μ_T_	*a*_sw_	*F*_sw_	*F*_sq_	*c*	κ

Gauss	1/16	0	6.30	6.30	0.91	0.333
	1/4	0.01	6.43	6.44	0.86	0.34
	1	0.25	8.00	8.40	0.56	0.46
	4	0.66	47.3	86	0.30	0.50

exponential	1/16	0	6.38	6.38	0.62	0.35
	1/4	0	6.89	6.89	0.68	0.44
	1	0	13.85	13.85	0.46	0.48
	4	0	339	339	0.28	0.49

**Figure 18:**
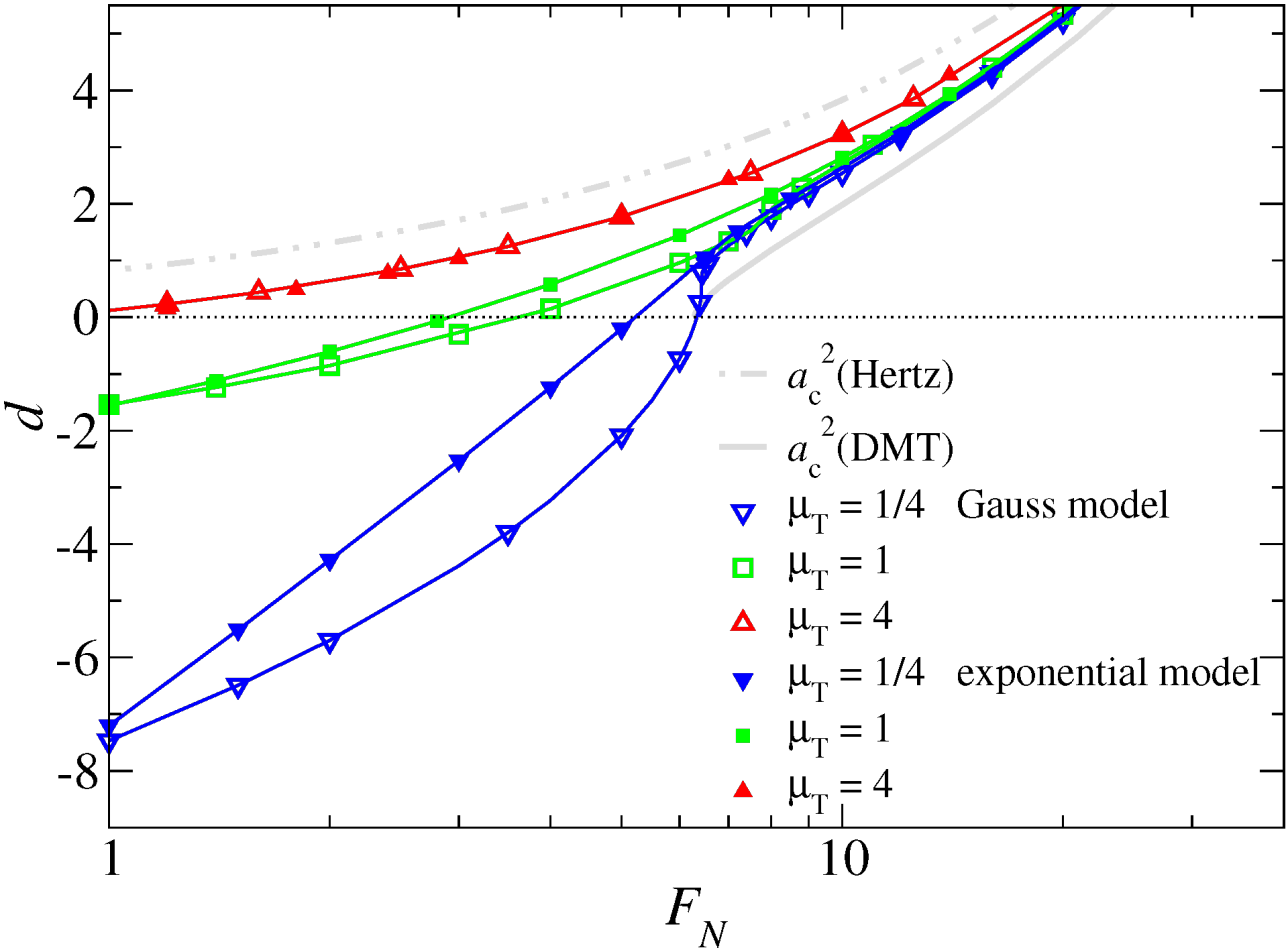
Displacement *d* as a function of normal force *F*_N_ in the vicinity of the spontaneous wetting force *F*_sw_. Symbols reflect numerical results. The lines, which connect many data points not explicitly shown, are drawn to guide the eye. The two thick grey lines reflect the square of the contact radius in the Hertz and DMT approximation, respectively. Color coding: μ_T_ = 4 (red), μ_T_ = 1 (green), and μ_T_ = 1/4 (blue).

As in the case of adhesive interactions, the normal displacement seems less sensitive to both the choice of the potential and the Tabor coefficient than the contact area, unless normal loads are very small, i.e., at loads similar in magnitude or smaller than the squeeze-out load for μ_T_ = 1. This is demonstrated in Figure 18. It reveals that information on the (effective) near-range surface interactions at small separation are difficult to obtain from experimentally measured load-displacement curves.

**Figure 19:**
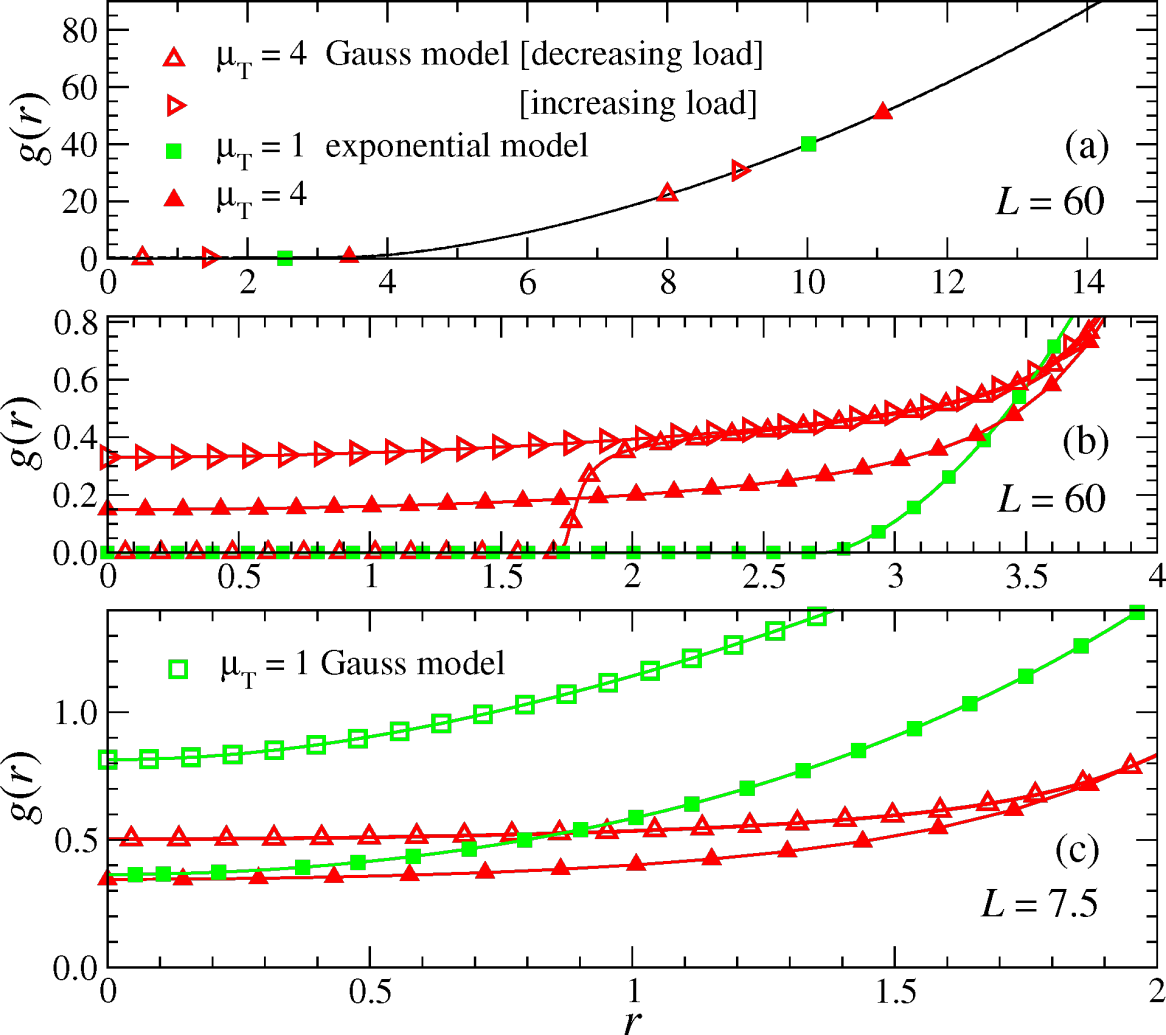
Gap *g*(*r*) as a function of the lateral distance from the origin *r* for a large load *F*_N_ = 60 (a) and (b) as well as for an intermediate load *F*_N_ = 7.5 (c). In each case, surfaces repel each other. Graph (b) contains the same data as (a) but has higher resolution. Color coding: μ_T_ = 4 (red) and μ_T_ = 1 (green).

I conclude this section with an analysis of the gap profile for repulsive contacts. At large loads, different Tabor parameters and functional forms for finite-range repulsion yield gap profiles that are indistinguishable at small magnification, see Figure 19a. Differences become nevertheless significant at high resolution near the center of the contact. Particularly remarkable is the data set for the Gauss model with μ_T_ = 4 and its bistability revealed in Figure 19b. For an increasing force, no contact has formed at *F*_N_ = 7.5. However, when reaching *F*_N_ = 7.5 from above, contact is formed for radii *r* < *a*_c_ ≈ 1.73. In the latter case, the gap then quickly increases within Δ*r* ≈ 0.1 to an almost constant value of order 1/μ_T_ for *r* ≥ *a*_c_, as if one had a single confined layer of liquid. For radii *r* > 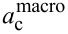, the gap assumes the “macroscopic” behavior. Here, 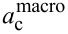 ≈ 4 is the contact radius that one would ascertain from the analysis of the gap with low resolution, e.g., via graphical inspection of Figure 19a.

At small loads, the sensitivity of the gap profile on the details of the model become even more apparent. This result, which can be seen in Figure 19c, is expected, since the elasticity of the tip is no longer relevant. Instead, the force-displacement curve is predominantly determined by the effective surface interactions, as shown clearly by the μ_T_ = 4 data sets in Figure 18. They exhibit, to leading order, a *F*_N_ 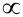 exp(−*d*/ζ) relation in the exponential model, and a *F*_N_ 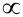 exp(−*d*^2^/ζ^2^) relation for the Gauss model, where ζ is inversely proportional to μ_T_.

## Conclusion

The principle new aspect of this work is the continuum-mechanics based analysis of single-asperity contacts with finite-range repulsion acting in addition to short-range hard-wall repulsion. The analysis is based on the concept of the Tabor coefficient and the repulsion is assumed to arise due to the presence of a strongly wetting fluid. As for attractive single-asperity contacts, it is found that the contact area or the displacement on the normal load depend, to a large degree, not only on the surface energy but also on the Tabor coefficient μ_T_. Moreover, for μ_T_ exceeding a critical value, there may exist a range of loads in which two (meta)stable solutions coexist, i.e., one in which the surfaces touch and one in which a thin gap between the two surfaces remains. When the value for the load is increased above a threshold, the latter solution becomes unstable and the gap disappears. However, in order to obtain this kind of behavior, which is reminiscent of the squeeze-out of a wetting fluid, the finite-range interactions between the contacting surfaces have to be tailored correctly. Using a surface interaction *v*_fr_, whose derivative increases monotonically as the gap *g* approaches zero, such as *v*_fr_ 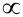 exp(−*g*/*z*_0_), only one stable solution exists for any given normal load. Conversely, when the distance–force dependence is multi-valued, as is the case for a *v*_fr_ 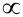 
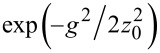
 relation, squeeze-out and spontaneous wetting can be rationalized and thus be modeled in the realm of continuum mechanics – in terms of transitions between (meta)stable solutions. These transitions (similar to instabilities in the Prandtl model [26], in which a particle is dragged with a weak spring through a sinusoidal potential) can occur for solvated tips on surfaces, for example, if the effective tip–surface interactions has zero slope when the surfaces touch, as is the case for *v*_fr_ 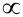 
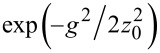
. In reality, the far-field potential may even be oscillatory and evidenced by the squeeze-out of many subsequent layers. Such behavior has been recently observed and linked to the (damped) long-range oscillatory behavior of the density correlations in high-density liquids [15,19].

**Figure 20:**
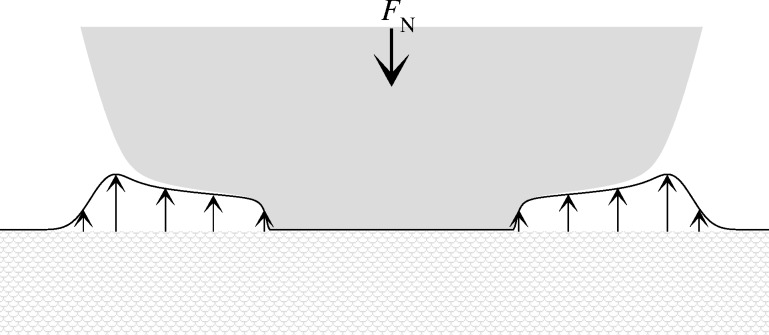
Contact geometry for a Gauss model with finite-range repulsion. Arrows indicate the direction of normal load *F*_N_ (thick arrow) and that of the finite-range repulsion (thin arrows) acting in addition to a hard-wall constraint. No adhesive forces between the surfaces are considered.

An interesting consequence of short-range repulsion is that the contact geometry can look similar to that of an adhesive neck. This is shown in Figure 19b for the (μ_T_ = 4) Gauss model and decreasing load. To improve the visualization, a similar gap geometry is shown again in Figure 20 together with a profile of the finite-range repulsion.

A secondary aspect of this work is devoted to the analysis of how to best reach well-defined asymptotic behavior in numerical simulations of adhesive contact mechanics. It is found that the DMT limit is approached quickest when using attractive potentials whose first derivative disappears as the gap goes to zero, at least if the contact area is the variable of interest. However, these potentials approach the JKR limit only at a rate of 1/μ_T_ for large μ_T_ and the contact area becomes difficult to define once μ_T_ ≥ 1. Thus, one is better off using potentials with finite slope in the small-gap limit. They converge in a well-defined fashion with 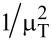 to the JKR limit for large Tabor coefficients. This is supposedly the more relevant limit for adhesive surfaces with self-affine fractal roughness. For the modeling of repulsive surfaces, the situation is more complicated. Formally, the JKR limit is again reached more quickly with models that have finite slope at zero gap. However, these models do not allow one to model the hysteretic response of a confined fluid that results whenever the squeeze-out force exceeds the spontaneous wetting force.

A by-product of this work is a minor modification of the phenomenological description of single-asperity contact mechanics by Carpick, Ogletree, and Salmeron [1]. The COS equations can be parametrized to contain the correct asymptotic behavior for JKR and for DMT limits and also for the superposition of extremely short- and long-range interfacial interactions, as shown by Schwarz [2]. However, they still have a few formal shortcomings for intermediate-range potentials. For example, the original interpolation of the contact-area-on-load dependence for finite Tabor coefficients recuperates neither Hertzian contact mechanics at large loads with correct prefactors nor the correct contact radii in either DMT or JKR limit at zero normal loads. In this work, I propose to enforce those limits exactly including the correct asymptotics for *a*_c_(µ_T_,*F*_N_ = 0) at μ_T_ = 0 and μ_T_ → ∞. By doing so, the maximum error of the *a*_c_(*F*_N_ = 0,μ_T_) curve could be reduced from 1.2% to less than 0.3%. A shortcoming of both the original and the new, modified COS equations is that they both assume an asymptotic behavior near pull-off (*F*_N_ → −*F*_p_) according to (*a* − *a*_p_) 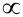 (*F*_p_ + *F*_N_)^κ^, where the exponent takes the JKR value κ = 2/3 for any non-zero Tabor coefficient. The modified COS equations could thus be improved further if one incorporated the new finding that κ crosses over continuously from 2/3 (exact for JKR) to 1/2 (exact for DMT). However, this does not seem useful in practice. Extreme accuracies (5 digits and more for *a*_c_ and *F*_N_) would be needed in measurements to deduce *a*_p_ and κ to within one or two digits. Such an accuracy is difficult to achieve both experimentally and numerically. Moreover, the surface energy is not very well defined at small scales, because its precise value depends crucially on roughness down to the atomic scale, see, e.g., [27]. Thus, from a practical point of view, both the original and the modified COS equations are quite reasonable, all the more because the geometry of real tips can deviate quite substantially from a parabola.

This work is concluded with an assessment of what values for μ_T_ one might expect in AFM or SFA experiments. To come up with a ballpark estimate, the following “typical values” shall be assumed: Δγ = 40 mN (Δγ can, of course, be close to zero, but much higher, e.g., for two equally charged surfaces in the context of electrochemistry), *E* = 5 GPa (in between soft matter and ceramics), *z*_0_ = 10 Å (size of an OMCTS or molten salt molecule), *R* = 1 μm (in between AFM and SFA, precise value not very important, as third root is taken). These numbers lead to μ_T_ = 0.4, which is close to the interesting “cross-over” regime. Thus, real contacts may span a broad range of values for μ_T_. Comparison between theory and experiment may be difficult, in particular because atomic-scale roughness (or even sub-atomic roughness arising from electron orbitals) leads to complicated slip-boundary conditions and slow kinetics. However, given a well-motivated form for the effective interaction between two flat surfaces, it may yet be possible to rationalize and to model, at least on a semi-quantitative level, the interactions of curved surfaces in the presence of a strongly wetting fluid within the presented Tabor-coefficient based framework. Particularly appealing systems may be found in tribo-electrochemical applications, where the surface interactions can be tailored in a quasi-continuous fashion.
